# A hybrid expectation maximisation and MCMC sampling algorithm to implement Bayesian mixture model based genomic prediction and QTL mapping

**DOI:** 10.1186/s12864-016-3082-7

**Published:** 2016-09-21

**Authors:** Tingting Wang, Yi-Ping Phoebe Chen, Phil J. Bowman, Michael E. Goddard, Ben J. Hayes

**Affiliations:** 1 School of Engineering and Mathematical Sciences, La Trobe University, Melbourne, VIC Australia; 2 Biosciences Research, Department of Economic Development, Jobs, Transport and Resources, Bundoora, Melbourne, VIC Australia; 3 Dairy Futures Cooperative Research Centre, Melbourne, VIC Australia; 4 School of Applied Systems Biology, La Trobe University, Melbourne, VIC Australia; 5 Faculty of Veterinary and Agricultural Science, University of Melbourne, Melbourne, VIC Australia; 6 Queensland Alliance for Agriculture and Food Innovation, Centre for Animal Science, University of Queensland, Queensland, Australia

## Abstract

**Background:**

Bayesian mixture models in which the effects of SNP are assumed to come from normal distributions with different variances are attractive for simultaneous genomic prediction and QTL mapping. These models are usually implemented with Monte Carlo Markov Chain (MCMC) sampling, which requires long compute times with large genomic data sets. Here, we present an efficient approach (termed HyB_BR), which is a hybrid of an Expectation-Maximisation algorithm, followed by a limited number of MCMC without the requirement for burn-in.

**Results:**

To test prediction accuracy from HyB_BR, dairy cattle and human disease trait data were used. In the dairy cattle data, there were four quantitative traits (milk volume, protein kg, fat% in milk and fertility) measured in 16,214 cattle from two breeds genotyped for 632,002 SNPs. Validation of genomic predictions was in a subset of cattle either from the reference set or in animals from a third breeds that were not in the reference set. In all cases, HyB_BR gave almost identical accuracies to Bayesian mixture models implemented with full MCMC, however computational time was reduced by up to 1/17 of that required by full MCMC. The SNPs with high posterior probability of a non-zero effect were also very similar between full MCMC and HyB_BR, with several known genes affecting milk production in this category, as well as some novel genes. HyB_BR was also applied to seven human diseases with 4890 individuals genotyped for around 300 K SNPs in a case/control design, from the Welcome Trust Case Control Consortium (WTCCC). In this data set, the results demonstrated again that HyB_BR performed as well as Bayesian mixture models with full MCMC for genomic predictions and genetic architecture inference while reducing the computational time from 45 h with full MCMC to 3 h with HyB_BR.

**Conclusions:**

The results for quantitative traits in cattle and disease in humans demonstrate that HyB_BR can perform equally well as Bayesian mixture models implemented with full MCMC in terms of prediction accuracy, but with up to 17 times faster than the full MCMC implementations. The HyB_BR algorithm makes simultaneous genomic prediction, QTL mapping and inference of genetic architecture feasible in large genomic data sets.

**Electronic supplementary material:**

The online version of this article (doi:10.1186/s12864-016-3082-7) contains supplementary material, which is available to authorized users.

## Background

Genomic prediction of genetic merit, using SNP markers, is now routinely used in animal and plant breeding to identify superior breeding individuals and so accelerate genetic gain [1–3]. Genomic prediction methodology is also increasingly used in human disease studies for the inference of genetic architecture, the identification of causal mutations (QTL mapping), and prediction of disease risk [4–8].

Genomic predictions are often implemented using linear prediction models, especially best linear unbiased prediction (BLUP) or Genomic BLUP (GBLUP), which assume that all the SNPs contribute small effects to the trait and these effects are derived from a normal distribution [1, 4, 9]. While BLUP based genomic predictions have certainly been successful in increasing genetic gain in livestock and crops [10, 11], this approach does have some limitations. One limitation is that the prediction accuracy does not persist well across multiple generations, because the severe shrinkage in these models results in the effect of causative mutation being “smeared” across many markers encompassing long chromosome segments – in other words a linear combination of effects of a large number of markers is used to capture the effect of a QTL. After several generations, the association between markers and QTL might be broken down by recombination, thereby reducing prediction accuracy. The smearing of effect of causative mutations across many SNP also results in imprecise QTL mapping with BLUP methods.

To address these problems, Bayesian mixture models (nonlinear prediction e.g. Bayes A, B, C, and R) [1, 6, 12–15] assume non-normal prior distributions of SNP effects. One example of a flexible approach, BayesR [14] defines a mixture model with SNP effects following a mixture of four normal distributions with zero, very small, small and moderate variances. In practice, the prediction accuracy of Bayesian mixture models (including BayesR) has been shown to be equal or superior to that of GBLUP for both human diseases and dairy cattle milk production traits [15–25].

In addition to the prediction of breeding values and future phenotypes using genotype data, Bayesian models (such as BayesR) can be used to map the causal polymorphisms (quantitative trait loci or QTL). For this purpose they have some advantages over traditional single SNP regression, which is commonly used to analyse genome wide association studies (GWAS) [16–24]. Single SNP regression fits one SNP at a time as a fixed effect and tests the significance of the association between the SNP and the trait, while ignoring all other SNPs. To protect against performing multiple tests, stringent P-values (*P* < 5*10^−8^) are used. This method of analysis has three limitations:1) The effect of those SNPs declared significant is usually over-estimated; 2) multiple SNPs in linkage disequilibrium with the same QTL may show an association with the trait leading to imprecision in mapping the QTL; 3) many QTL are not detected at all because no SNP reaches the stringent *P*-value for association with the trait. By comparison, Bayesian mixture models fit all SNPs simultaneously by treating the SNP effects as random effects drawn from a prior distribution. For example, the BayesR model has been implemented for QTL detection in the dairy cattle genome and for human disease traits [15]. The results show that BayesR can increase the power of identifying the known genes in contrast with GBLUP and GWAS.

Even though nonlinear models are attractive, one limitation is that nonlinear models typically require long computational times. Due to the hierarchical estimation of posterior distributions of SNP effects and their variances, nonlinear models have usually been implemented with Markov Chain Monte Carlo (MCMC). This requires a large number of iterations with time per iteration scaled linearly with the number of markers (*m*) and the number of individuals (*n*). Genomic data sets are now often very large and are rapidly becoming larger. For human, 300,000 to 9 million SNPs arrays genotyped on up to 253 K individuals [26, 27] are available for association studies and disease/fitness prediction. In dairy cattle, whole genome sequence data including 39 million variants has been published by the 1000 bull genomes project [28]. When confronted with such huge genomic data, Bayesian methods can be so computationally expensive that it is not possible to use them.

Two approaches have been used to develop more computationally efficient algorithms for implementing Bayesian mixture models. One is to modify MCMC with speed-up schemes. For example, Moser et al. [8] introduced a “500SNPs” scheme to pick 500 SNPs with non-zero effects to be updated instead of all the SNPs. Such modification schemes could reduce the running time by 3 ~ 6 fold. Calus et al. [29] proposed a right-hand-side updating algorithm to cluster multiple SNPs (similar to a haplotype) to be updated as one during MCMC iterations. The results on 50 K SNP panels demonstrated up to 90 % reduction in computational time without reducing prediction accuracy. The other approach is to introduce heuristic methods (e.g. iterated conditional expectation, ICE; expectation maximisation, EM) as an alternative to MCMC. There are a wide range of fast versions of Bayesian approaches to genomic prediction using these methods (including fastBayesB, emBayesB, emBayesR) [30–35], which are several orders faster than MCMC implementations. However, none of these algorithms gives consistently as high prediction accuracy as their MCMC counterparts. The EM method of Wang et al. [30], emBayesR, gave higher accuracy than ICE based methods but still had a reduction in accuracy of 5 % ~ 7 % for traits with mutations of moderate to large effect. In other words, the heuristic approximations works best when there are no mutations of moderate to large effect, otherwise accuracy can be compromised. This is undesirable, especially when the largest advantage of the non-linear Bayesian methods over BLUP is observed when there are mutations of moderate to large effect (where moderate effect can mean a QTL explaining 1 % of the variance if the data set is large)!

Motivated by the deficiency of both MCMC (long computing terms) and fast versions of nonlinear models (lower prediction accuracy with some genetic architectures), we hypothesise that a hybrid scheme, beginning with EM iterations and finishing with MCMC sampling iterations, would give similar prediction accuracy to a full MCMC implementation, while having a significant speed advantage. Here we propose a hybrid algorithm (termed HyB_BR) of Expectation-Maximisation (EM) (emBayesR) and MCMC under the BayesR model. The algorithm also incorporates a speed-up scheme where only a proportion of SNP continue to be sampled in MCMC iterations. In comparison with emBayesR [30], the main improvement is that HyB_BR introduces a limited number of MCMC iterations after EM to improve the solutions from emBayesR.

To evaluate the predictive ability and computational efficiency of HyB_BR, prediction accuracy was compared with BayesR and GBLUP in two data sets. The first data set was 800 K SNP genotypes in 16,214 Holstein and Jersey bulls and cows. The prediction accuracy within these breeds and in a third breed (Aussie Red) was evaluated. The results showed that HyB_BR achieved very similar prediction accuracy to BayesR, while reducing the running time by up to 17 fold, and overcoming the limitations of slightly reduced accuracy of emBayesR. As a result of running the algorithm, the posterior probability of each SNP being in the model was derived, and this was used for QTL mapping. The resulting QTL regions were compared between the approaches and with previous literature reports. The results demonstrated that HyB_BR has enough power to detect the major known genes affecting milk production traits in dairy cattle as well as some novel regions. HyB_BR was also evaluated in a second data set - the Welcome Trust Case Control Consortium (WTCCC) human disease data set [27]. The results demonstrated that HyB_BR is a promising method for risk prediction and genetic architecture inference for human disease traits as well.

## Methods

### The mixture data model

The overall model at the level of the data for HyB_BR (independent of MCMC and EM implementation) including all the relevant parameters and priors is described first. The model assumes that **y**, the phenotypic records of *n* individuals, is a linear model of fixed effects (**β**), SNP effect (**g**), random polygenic effects (**v**) and environmental errors (**e**):

1 $$ \mathbf{y}=\mathbf{X}\boldsymbol{\upbeta } +\mathbf{Zg}+\mathbf{W}\mathbf{v}+\mathbf{e}, $$

where,

**β** = vector of *p* fixed effects, following uninformative priors.

**g** = vector of *m* SNP effects. For each SNP, ${g}_i\sim b\left(i,1\right)\times N\left(0,0*{\sigma}_g^2\right)+b\left(i,2\right)\times N\left(0,0.0001*{\sigma}_g^2\right)+b\left(i,3\right)\times N\left(0,0.001*{\sigma}_g^2\right)+b\left(i,4\right)\times N\left(0,0.01*{\sigma}_g^2\right),$in which *σ*_*g*_^2^ is the genetic variance of the trait and *b*(*i*, *k*) is a scalar with two possible values {0, 1}, determining whether or not the effect of the *i*^*th*^ SNP is derived from the *k*^*th*^ distribution.

**Pr** = vector of probabilities where ***Pr***_*k*_ = scalar with the value range between 0 and 1. The parameter **Pr** defines the proportion of all the SNPs following each of four normal distributions *k*. ***Pr***_*k*_ is assumed to follow a Dirichlet distribution with the parameter α = (1, 1, 1, 1)^T^.

**v** = vector of *q* polygenic effects (breeding values, for the proportion of variance not explained by the SNP), with **v** ~ *N*(0, **A***σ*_*a*_^2^), **A** is the *q* × *q* pedigree-based relationship matrix, *σ*_*a*_^2^ is the polygenic variance, *q* is the number of individuals in the pedigree.

**e** = vector of *n* residual errors. For cattle data, **e** ~ *N*(0, **E**σ_*e*_^2^), where **E** is a *n* × *n* diagonal matrix so that the error variance associated with different records can vary. For example, for bulls, the phenotype would be daughter yield deviations, which would have a lower error variance than the trait deviations (TD) of cows [36]. For human data where all phenotypes have the same magnitude of error, **E** matrix can be replaced by the identity matrix **I**.

**X** = *n* × *p* design matrix, allocating phenotypes **y** to fixed effects **β**.

**Z** = *a n* × *m* genotype matrix with elements ${\mathbf{z}}_{\mathrm{ij}}=\left({\mathbf{s}}_{\mathrm{ij}}-2{p}_i\right)/\sqrt[2]{2{p}_i\left(1-{p}_i\right)}$, in which **s**_ij_ is the genotypes of the *j*^*th*^ individual for the *i*^*th*^ SNP (0, 1 or 2 copies of the second allele), and *p*_*i*_ is the allele frequency of each SNP *i*.

**W** = *n* × *q* design matrix, aims at allocating the *q* × 1 vector of polygenic effects to **y**.

Note that model (1) extends the model used by Wang et al. [30] to include fixed effects, polygenic effects and weights.

The prior distribution of each SNP effect *g*_*i*_ conditional on *b*(*i*, *k*) is

$$ \mathrm{p}\left({g}_i\Big|b\left(i,k\right)\right)=\left\{\begin{array}{c}\hfill \delta \left({g}_i\right),\ b\left(i,1\right)=1\ \hfill \\ {}\hfill \frac{1}{\sqrt{2\pi {\upsigma}_{\mathrm{i}}^2\left[k\right]}} \exp \left(-\frac{g_i^2}{2{\upsigma}_{\mathrm{i}}^2\left[k\right]}\right),\ b\left(i,k\right)=1\left(k=2,3,4\right)\hfill \end{array}\right.\ . $$

Where, δ(g_i_) denotes the Dirac delta function with all probability mass at g_i_ = 0.

The joint distribution p(*g*_*i*_, *b*(*i*, *k*)|*Pr*_*k*_) (i.e. conditional on *Pr*_*k*_) can be written as:

2 $$ \begin{array}{l}p\left({g}_i,b\left(i,k\right)\Big|P{r}_k\right)={\displaystyle {\prod}_{k=1}^4p}\left({g}_i\Big|b\left(i,k\right)\right)\times p\left(b\left(i,k\right)\Big|P{r}_k\right)\\ {}\kern3.5em ={\left(\updelta \left({g}_i\right)P{r}_1\right)}^{b\left(i,1\right)}{{\displaystyle {\prod}_{k=2}^4\left(\frac{1}{\sqrt{2{\uppi \upsigma}_{\mathrm{i}}^2\left[k\right]}} \exp \left(-\frac{g_i^2}{2{\upsigma}_{\mathrm{i}}^2\left[k\right]}\right)P{r}_k\right)}}^{b\left(i,k\right)}\end{array} $$

The implementation of HyB_BR with the mixture model defined above consists of two components: 1) The Expectation-Maximization module. HyB_BR first implements the EM iterations under the mixture Gaussian model (Eq. 2) to give approximations for the parameter set including SNP effects **g**, proportion of SNP in each distribution **Pr**, error variance σ_*e*_^2^, and polygenic variance *σ*_*a*_^2^. For the estimation of each SNP effect, the PEV (predicted error variance) correction is introduced to account for the errors which are generated from the estimations of all other SNP effects (detailed in Additional file 1). 2) MCMC module. Once the EM steps are converged, the output values of the parameters are used in the modified MCMC iterations as the start values. For the final step, a MCMC scheme is implemented with a limited number of iterations.

### EM module

In the following EM modules, the parameter set *θ* = {**g**, **Pr**, **β**, **v**, σ_*e*_^2^} will be estimated by their maximum a posteriori (MAP) value. Similar to emBayesR [30], all the parameters *θ* were estimated according to the expectation-maximisation process with steps:

i) Define the log likelihood *f*(**y**|*θ*) of the data under the data model (1).
ii) Derive the log posterior function of the parameters using Bayes’ theorem. Following Bayes’ theorem, the log posterior distribution of the parameter sets *θ* is based on the rule *logp*(*θ*|**y**) ∝ *logf*(**y**|*θ*) + *logp*(*θ*), with *p*(*θ*) the prior for the parameter.
iii) Take the expectation on the posterior function over the missing data.
iv) Differentiate the expected posterior function and solve for this equal to zero to obtain MAP (Maximum A Posterior) of the parameter set *θ*.

In the Expectation-maximization implementation, the posterior distributions for each parameter *p*(*θ*|**y**) are obtained while “integrating out” the other parameters. For example, for the estimation of each SNP effect *g*_*i*_ (SNP *i* in the vector **g**), we maximize the posterior distribution of each SNP effect *p*(*g*_*i*_|**y**, *b*(*i*, *k*), Pr_k_, **β**, **v**, σ_*e*_^2^) by integrating out the other SNP effects *g*_*j* ≠ *i*_, the parameters *b*(*i*, *k*), **β**, **v**, but we fix the proportion parameter Pr_k_ and the error variance σ_*e*_^2^ at their maximum posterior estimates. In the following, we will detail the inference process for several key parameters including SNP effects (**g**), the mixing parameters (*Pr*_*k*_), fixed effects (**β**), polygenic effects (**v**) and the error variance (*σ*_*e*_^2^) separately:

1. **Estimation of SNP effects g**

As in our EM version of BayesR [30], in HyB_BR we will update the estimated effect of SNPs one at a time. Therefore, we rewrite **Zg** in Eq. (1) as the sum of the effect of the current SNP **Z**_**i**_*g*_*i*_ and the combined effect of all other SNP effects **u**_***i***_ (**u**_***i***_ = ∑_***j*** ≠ ***i***_**Z**_**j**_*g*_*j*_). We rewrite the model (1) as:

3 $$ \mathbf{y}=\mathbf{X}\boldsymbol{\upbeta } +{\mathbf{Z}}_{\mathbf{i}}{g}_i+{\mathbf{u}}_{\boldsymbol{i}}+\mathbf{W}\mathbf{v}+\mathbf{e} $$

where, *g*_*i*_(the effect of SNP *i*) is the *i*^*th*^ element of the vector **g**, and **u**_***i***_ = ∑_***j*** ≠ ***i***_**Z**_**j**_*g*_*j*_.

We estimate of *ĝ*_*i*_ by the value of *g*_*i*_ that maximises the posterior probability $\mathrm{P}\left({g}_i\Big|\mathbf{y},\widehat{\mathbf{P}}\mathbf{r},\widehat{\upsigma_e^2}\right)$ where $\widehat{\mathbf{P}}\mathbf{r}$ and $\widehat{\upsigma_e^2}$ are the MAP estimates of **Pr** and σ_*e*_^2^ conditional on **y**.

To perform this, we first introduce “missing data” (*b*(*i*, *k*), **β**, **v**, **u**_***i***_), and then “integrate them out” via the Expectation-Maximisation steps. In detail, the marginal posterior distribution of each SNP effect *g*_*i*_ can be written as:

$$ p\left({g}_i,b\left(i,k\right)\Big|\mathbf{y},\boldsymbol{\upbeta}, \mathbf{v},{\mathbf{u}}_{\boldsymbol{i}},\widehat{\upsigma_{\mathrm{e}}^2},\ {\widehat{Pr}}_k\right)\propto p\left(\mathbf{y}\Big|{g}_i,b\left(i,k\right),\boldsymbol{\upbeta}, \mathbf{v},{\mathbf{u}}_{\boldsymbol{i}},\widehat{\upsigma_{\mathrm{e}}^2},\ {\widehat{Pr}}_k\right)p\left({g}_i,b\left(i,k\right)\Big|{\widehat{Pr}}_k\right). $$

Under the model (3), the first term $p\left(\mathbf{y}\Big|{g}_i,b\left(i,k\right),\boldsymbol{\upbeta}, \mathbf{v},{\mathbf{u}}_{\boldsymbol{i}},\widehat{\upsigma_{\mathrm{e}}^2},\ {\widehat{Pr}}_k\right)$ is obtained according to the following normal distribution:

**y** − X**β** − **Z**_**i**_*g*_*i*_ − **Wv** − **u**_***i***_ ~ *N*(0, **E**σ_e_^2^), 

which can be transformed as:

$$ {\mathbf{e}}^{*}-{\mathbf{Z}}_{\mathbf{i}}{g}_i\sim N\left(0,\ \mathbf{E}{\upsigma}_{\mathrm{e}}^2\right), $$

Where, e* = **y** − X**β** − **Wv** − **u**_***i***_.

Therefore, the term $p\left(\mathbf{y}\Big|{g}_i,b\left(i,k\right),\boldsymbol{\upbeta}, \mathbf{v},{\mathbf{u}}_{\boldsymbol{i}},\widehat{\upsigma_{\mathrm{e}}^2},\ {\widehat{Pr}}_k\right)$ can be written as: $p\left(\mathbf{y}\Big|{g}_i,{\mathbf{u}}_{\boldsymbol{i}},b\left(i,k\right),\boldsymbol{\upbeta}, \mathbf{v},\widehat{\upsigma_{\mathrm{e}}^2},\ {\widehat{Pr}}_k\right)=\frac{1}{{\left(2\pi \widehat{\sigma_e^2}\right)}^{\frac{n}{2}}}\frac{1}{\left|\mathbf{E}\right|} exp\left[-\frac{1}{2\widehat{\sigma_e^2}}\left({\mathbf{e}}^{*}-{\mathbf{Z}}_{\mathrm{i}}{g}_i\right)\mathit{\hbox{'}}\;{\mathbf{E}}^{-1}\left({\mathbf{e}}^{*}-{\mathbf{Z}}_{\mathrm{i}}{g}_i\right)\right]$

Then the log likelihood function is:

4 $$ logp\left(\mathbf{y}\Big|{g}_i,{\mathbf{u}}_{\boldsymbol{i}},b\left(i,k\right),\boldsymbol{\upbeta}, \mathbf{v},\widehat{\upsigma_{\mathrm{e}}^2},\ {\widehat{Pr}}_k\right)=-\frac{\mathrm{n}}{2} \log \widehat{\upsigma_{\mathrm{e}}^2}- \log \left|\mathbf{E}\right|-\frac{1}{2\widehat{\upsigma_{\mathrm{e}}^2}}\left({\mathbf{e}}^{*}-{\mathbf{Z}}_{\mathrm{i}}{g}_i\right)\mathit{\hbox{'}}\;{\mathbf{E}}^{-1}\left({\mathbf{e}}^{*}-{\mathbf{Z}}_{\mathrm{i}}{g}_i\right) $$

Ignoring an additive constant, the second term $p\left({g}_i,b\left(i,k\right)\Big|{\widehat{Pr}}_k\right)$ is defined in the Eq. (2). Then the log of $p\left({g}_i,b\left(i,k\right)\Big|{\widehat{Pr}}_k\right)$ is:

5 $$ \begin{array}{l} logp\left({g}_i,b\left(i,k\right)\Big|{\widehat{Pr}}_k\right)=b\left(i,1\right) \log \left(\delta \left({g}_i\right){\widehat{Pr}}_1\right)\\ {}\kern15em +{\displaystyle {\sum}_{\mathrm{k}=2}^4b}\left(i,k\right)\left(-\frac{1}{2}{ \log \upsigma}_i^2\left[k\right]-\frac{g_i^2}{2{\upsigma}_i^2\left[k\right]}+ \log {\widehat{Pr}}_{\mathrm{k}}\right)\end{array} $$

Treating (e*, *b*(*i*, *k*)) as missing data and omitting the terms without *g*_*i*_, the expectation of the log marginal posterior of each SNP effect is:

$$ \begin{array}{l}{E}_{{\mathbf{e}}^{*},b\left(i,k\right)} logp\left({g}_i,b\left(i,k\right)\Big|\mathbf{y},\boldsymbol{\upbeta}, \mathbf{v},{\mathbf{u}}_{\boldsymbol{i}},\widehat{\upsigma_{\mathrm{e}}^2},\ {\widehat{Pr}}_k\right)\\ {}={E}_{{\mathbf{e}}^{*},b\left(i,k\right)} logp\left(\mathbf{y}\Big|{g}_i,{\mathbf{u}}_{\boldsymbol{i}},b\left(i,k\right),\boldsymbol{\upbeta}, \mathbf{v},\widehat{\upsigma_{\mathrm{e}}^2},\ {\widehat{Pr}}_k\right)+{E}_{{\mathbf{e}}^{*},b\left(i,k\right)} logp\left({g}_i,b\left(i,k\right)\Big|{\widehat{Pr}}_k\right)\end{array} $$

According to Eq (4), the expectation of the first term is:

6 $$ \begin{array}{l}{E}_{{\mathbf{e}}^{*},b\left(i,k\right)} logp\left(\mathbf{y}\Big|{g}_i,{\mathbf{u}}_{\boldsymbol{i}},b\left(i,k\right),\boldsymbol{\upbeta}, \mathbf{v},\widehat{\upsigma_{\mathrm{e}}^2},\ {\widehat{Pr}}_k\right)\kern0.5em \\ {}\kern3em \propto -\frac{1}{2\widehat{\upsigma_{\mathrm{e}}^2}}\left\{\left({\mathbf{e}}^{*}-{\mathbf{Z}}_{\mathrm{i}}{\mathrm{g}}_{\mathrm{i}}\right)\mathit{\hbox{'}}\;{\mathbf{E}}^{-1}\left({\mathbf{e}}^{*}-{\mathbf{Z}}_{\mathrm{i}}{\mathrm{g}}_{\mathrm{i}}\right)+tr\left({\mathbf{E}}^{-1}\mathrm{P}\mathrm{E}\mathrm{V}\left({\mathbf{e}}^{*}\right)\right)\right\}\end{array} $$

According to the Eq. (5), the expectation of the second term is:

7 $$ \begin{array}{l}{E}_{{\mathbf{e}}^{*},b\left(i,k\right)} logp\left({g}_i,b\left(i,k\right)\Big|{\widehat{Pr}}_k\right)\\ {}\propto P\left(i,1\right) \log \left(\delta \left({g}_i\right){\widehat{Pr}}_1\right)+{\displaystyle {\sum}_{\mathrm{k}=2}^4P}\left(i,k\right)\left(-\frac{1}{2}{ \log \upsigma}_i^2\left[k\right]-\frac{g_i^2}{2{\upsigma}_i^2\left[k\right]}+ \log {\widehat{Pr}}_{\mathrm{k}}\right)\end{array} $$

Where, $P\left(i,k\right)=E\left(b\left(i,k\right)\Big|\mathbf{y},\ {\widehat{Pr}}_k\right)$. The term *P*(*i*, *k*) can be derived as in the Additional file 2.

Hence, in the Maximisation steps of EM, we differentiate Eqs. (6) and (7) with respect to *ĝ*_*i*_, and then obtain an estimate for the SNP effect as:

8 $$ \begin{array}{l}\frac{\partial {E}_{{\mathbf{e}}^{*},b\left(i,k\right)} logp\left({g}_i,{\mathbf{u}}_{\boldsymbol{i}},b\left(i,k\right)\Big|\mathbf{y},\widehat{\boldsymbol{\upbeta}},\widehat{\mathbf{v}},\widehat{\upsigma_{\mathrm{e}}^2},\ {\widehat{Pr}}_k\right)}{\partial {\mathrm{g}}_i}=\left[-{\displaystyle \sum_{\mathrm{k}=2}^4\frac{P\left(i,k\right)}{\upsigma_i^2\left[k\right]}}-\frac{{\mathbf{Z}}_{\mathrm{i}}^{\mathit{\hbox{'}}}{\mathbf{E}}^{-1}{\mathbf{Z}}_{\mathbf{i}}}{\widehat{\upsigma_{\mathrm{e}}^2}}\right]{g}_i+\frac{{\mathbf{Z}}^{\mathit{\hbox{'}}}{\mathbf{E}}^{-1}{\mathbf{e}}^{*}}{\widehat{\upsigma_{\mathrm{e}}^2}}=0\\ {}{\widehat{g}}_i={\left[{\mathbf{Z}}_{\mathrm{i}}^{\mathit{\hbox{'}}}{\mathbf{E}}^{-1}{\mathbf{Z}}_{\mathrm{i}}+{\displaystyle {\sum}_{k=1}^4\left(P\left(i,k\right)\widehat{\upsigma_{\mathrm{e}}^2}{\upsigma}_i^2\left[k\right]\right)}\right]}^{-1}\left[{\mathbf{Z}}^{\mathit{\hbox{'}}}{\mathbf{E}}^{-1}{\mathbf{e}}^{*}\right]\end{array} $$

2) **Estimation of parameter Pr**

This follows a common method for an EM algorithm to analyse a mixture of distributions. We introduce the ‘missing data’ *b*(*i*, *k*) which is the indicator variable that indicates which of the *k* = 4 distributions SNP effect *i* is drawn from. The posterior distribution of proportion parameter **Pr** is:

$$ p\left(\mathbf{Pr},\mathbf{b}\Big|\mathbf{y}\right)\propto p\left(\mathbf{y}\Big|\mathbf{b}\right)p\left(\mathbf{b}\Big|\mathbf{Pr}\right)p\left(\mathbf{Pr}\right) $$

Where,

The term *p*(**y**|**b**) does not involve **Pr**. So when we differentiate with respect to **Pr**, this term will drop out and therefore can be ignored.

$$ \begin{array}{l}p\left(\mathbf{b}\Big|\mathbf{Pr}\right)={\displaystyle {\prod}_{i=1}^n{\displaystyle {\prod}_{k=1}^4{\left(P{r}_k\right)}^{b\left(i,k\right)}}}\\ {}p\left(\mathbf{Pr}\right)=\kern0.5em {\displaystyle {\prod}_{k=1}^4P{r}_k}\kern1em \end{array} $$

Therefore, the log posterior expression of **Pr** can be written as:

$$ \begin{array}{l} logp\left(\mathbf{Pr},\mathbf{b}\Big|\mathbf{y}\right)\propto logp\left(\mathbf{b}\Big|\mathbf{Pr}\right)+ logp\left(\mathbf{Pr}\right)\\ {}\kern11em ={\displaystyle {\sum}_{i=1}^n{\displaystyle {\sum}_{k=1}^4b}}\left(i,k\right) logP{r}_k+{\displaystyle {\sum}_{k=1}^4 logP{r}_k.}\end{array} $$

Treating **b** as the missing data and defining *P*(*i*, *k*) = *E*(*b*(*i*, *k*)|**y**, *Pr*_*k*_), the expectation of the posterior can be written as:

9 $$ {E}_{\mathbf{b}\Big|\mathbf{y}} logp\left(\mathbf{Pr},\mathbf{b}\Big|\mathbf{y}\right)={\displaystyle {\sum}_{i=1}^n{\displaystyle {\sum}_{k=1}^4P}}\left(i,k\right) logP{r}_k+{\displaystyle {\sum}_{k=1}^4 logP{r}_k.} $$

Introducing Lagrange multiplier *λ* to account for the constraint that ∑_*k* = 1_^4^*Pr*_*k*_ = 1 and differentiate with respect to *Pr*_*k*_, the parameter **Pr** can be estimated by:

10 $$ \begin{array}{l}\frac{\partial {\mathrm{E}}_{\mathbf{b}\Big|\mathbf{y}}\left[ logp\left(\mathbf{Pr},\mathbf{b}\Big|\mathbf{y}\right)+\uplambda \left({\displaystyle {\sum}_{\mathrm{k}=1}^4}P{r}_k-1\right)\right]}{\partial P{r}_k}=\frac{{\displaystyle {\sum}_{i=1}^m}P\left(i,k\right)}{P{r}_k}+\frac{1}{P{r}_k}+\lambda =0\\ {}\kern8.5em P{r}_k=\frac{{\displaystyle {\sum}_{\mathrm{i}=1}^m}P\left(i,k\right)+1}{{\displaystyle {\sum}_{k=1}^4}\left({\displaystyle {\sum}_{i=1}^m}\kern0.5em P\left(i,k\right)+1\right)}\end{array} $$

The computation of *P*(*i*, *k*) is given in the Additional file 2.

3) **Estimation of fixed effects (β) and the error variance (σ**_**e**_^2^**)**

Since fixed effects (**β**) and the error variance has uninformative priors, their posterior distribution is:

$$ p\left({\sigma}_e^2,\boldsymbol{\upbeta}, \widehat{\mathbf{g}}\Big|\mathbf{y}\right)=\frac{1}{{\left(2\pi {\sigma}_e^2\right)}^{\frac{n}{2}}}\frac{1}{\left|\mathbf{E}\right|} exp\left[-\frac{1}{2{\sigma}_e^2}\left(\mathbf{y}-\mathbf{Z}\widehat{\mathbf{g}}-\mathbf{X}\boldsymbol{\upbeta } -\mathbf{W}\widehat{\mathbf{v}}\right)\mathit{\hbox{'}}\;{\mathbf{E}}^{-1}\left(\mathbf{y}-\mathbf{Z}\widehat{\mathbf{g}}-\mathbf{X}\boldsymbol{\upbeta } -\mathbf{W}\widehat{\mathbf{v}}\right)\right] $$

As $\mathbf{y}-\mathbf{Z}\widehat{\mathbf{g}}-\mathbf{X}\boldsymbol{\upbeta } -\mathbf{W}\widehat{\mathbf{v}}=\mathbf{e}$, the full log likelihood based on this model is:

11 $$ logp\left({\sigma}_e^2,\boldsymbol{\upbeta}, \widehat{\mathbf{g}}\Big|\mathbf{y}\right)=-\frac{\mathrm{n}}{2}{ \log \upsigma}_{\mathrm{e}}^2+\frac{1}{2{\upsigma}_{\mathrm{e}}^2}\mathbf{e}\mathit{\hbox{'}}\;{\mathbf{E}}^{-1}\mathbf{e} $$

Treating **e** as the missing data, the expectation of the Eq. (11) can be expressed as

$$ \begin{array}{l}{\mathrm{E}}_{\mathbf{e}\Big|\mathbf{y}} logp\left({\sigma}_e^2,\boldsymbol{\upbeta}, \widehat{\mathbf{g}}\Big|\mathbf{y}\right)\\ {}={\mathrm{E}}_{\mathbf{e}\Big|\mathbf{y}}\left[-\frac{\mathrm{n}}{2}{ \log \upsigma}_{\mathrm{e}}^2+\frac{1}{2{\upsigma}_{\mathrm{e}}^2}\mathbf{e}\mathit{\hbox{'}}\;{\mathbf{E}}^{-1}\mathbf{e}\right]\\ {}=-\frac{\mathrm{n}}{2}{ \log \upsigma}_{\mathrm{e}}^2+\frac{1}{2{\upsigma}_{\mathrm{e}}^2}\left[\mathbf{e}\mathit{\hbox{'}}\;{\mathbf{E}}^{-1}\mathbf{e}+tr\left({\mathbf{E}}^{-1}\mathrm{P}\mathrm{E}\mathrm{V}\left(\mathbf{e}\right)\right)\right]\end{array} $$

In theory, PEV(**e**) ≠ PEV(e*) due to **e** = e* + **Z**_i_*g*_*i*_. However, since each SNP effect is shrunk severely towards zero by GBLUP [4], we approximate PEV(**e**) ≅ PEV(e*). The calculation of the term PEV(e*) is detailed in the Additional file 1.

Therefore, differentiating the equation E_**e**|**y**_*logp*(*σ*_*e*_^2^, **β**, **ĝ**|**y**) with regard to σ_e_^2^ and **b**, we achieve:

12 $$ \widehat{\upsigma_{\mathrm{e}}^2}=\frac{1}{\mathrm{n}}\left[\left(\mathbf{y}-\mathbf{Z}\widehat{\mathbf{g}}-\mathbf{X}\boldsymbol{\upbeta } -\mathbf{W}\widehat{\mathbf{v}}\right)\mathit{\hbox{'}}\;{\mathbf{E}}^{-1}\left(\mathbf{y}-\mathbf{Z}\widehat{\mathbf{g}}-\mathbf{X}\boldsymbol{\upbeta } -\mathbf{W}\widehat{\mathbf{v}}\right)+tr\left({\mathbf{E}}^{-1}\mathrm{P}\mathrm{E}\mathrm{V}\left({\mathbf{e}}^{*}\right)\right)\right] $$

13 $$ \widehat{\boldsymbol{\upbeta}}={\left(\mathbf{X}\mathit{\hbox{'}}\;{\mathbf{E}}^{-1}\mathbf{X}\right)}^{-1}\mathbf{X}\mathit{\hbox{'}}\;{\mathbf{E}}^{-1}\left(\mathbf{y}-\mathbf{Z}\widehat{\mathbf{g}}-\mathbf{W}\widehat{\mathbf{v}}\right) $$

4) **Estimation of polygenic effects (v)**

Under the model (1), the conditional posterior density function of polygenic effects **v** is:

$$ p\left(\mathbf{v}\Big|\mathbf{y}\right)=p\left(\mathbf{y}\Big|\mathbf{v},\widehat{\mathbf{g}},\widehat{\boldsymbol{\upbeta}},\widehat{\sigma_e^2}\right)p\left(\mathbf{v}\right) $$

Where,

14 $$ p\left(\mathbf{y}\Big|\mathbf{v},\widehat{\mathbf{g}},\widehat{\boldsymbol{\upbeta}},\widehat{\sigma_e^2}\right)=\frac{1}{{\left(2\pi {\sigma}_e^2\right)}^{\frac{n}{2}}}\frac{1}{\left|\mathbf{E}\right|} exp\left[-\frac{1}{2{\sigma}_e^2}\left(\mathbf{y}-\mathbf{Z}\widehat{\mathbf{g}}-\mathbf{X}\widehat{\mathbf{g}}-\mathbf{W}\mathbf{v}\right)\mathit{\hbox{'}}\;{\mathbf{E}}^{-1}\left(\mathbf{y}-\mathbf{Z}\widehat{\mathbf{g}}-\mathbf{X}\widehat{\boldsymbol{\upbeta}}-\mathbf{W}\mathbf{v}\right)\right] $$

15 $$ p\left(\mathbf{v}\right)=\frac{1}{{\left(2\pi {\upsigma}_{\mathrm{a}}^2\right)}^{\frac{q}{2}}}\frac{1}{\left|\mathbf{A}\right|} exp\left[-\frac{1}{2{\sigma}_a^2}\mathbf{v}\mathit{\hbox{'}}\;{\mathbf{A}}^{-1}\mathbf{v}\right] $$

Therefore, the log posterior based on Eqs. (14) and (15) is:

16 $$ \begin{array}{l} logp\left(\mathbf{v}\Big|\mathbf{y}\right)= logf\left(\mathbf{y}\Big|\mathbf{v},\widehat{\mathbf{g}},\widehat{\boldsymbol{\upbeta}},\widehat{\sigma_e^2}\right)+ logp\left(\mathbf{v}\right)\\ {}=\left[-\frac{\mathrm{n}}{2} \log \widehat{\upsigma_{\mathrm{e}}^2}- \log \left|\mathbf{E}\right|+\frac{1}{2\widehat{\upsigma_{\mathrm{e}}^2}}\left(\mathbf{y}-\mathbf{Z}\widehat{\mathbf{g}}-\mathbf{X}\widehat{\boldsymbol{\upbeta}}-\mathbf{W}\mathbf{v}\right)\mathit{\hbox{'}}\;{\mathbf{E}}^{-1}\left(\mathbf{y}-\mathbf{Z}\widehat{\mathbf{g}}-\mathbf{X}\widehat{\boldsymbol{\upbeta}}-\mathbf{W}\mathbf{v}\right)\right]\\ {}+\left[-\frac{\mathrm{q}}{2}{ \log \upsigma}_{\mathrm{a}}^2- \log \left|\mathbf{A}\right|+\frac{1}{2{\upsigma}_{\mathrm{a}}^2}\mathbf{v}\mathit{\hbox{'}}\;{\mathbf{A}}^{-1}\mathbf{v}\right]\end{array} $$

According to the equation $\mathbf{y}-\mathbf{Z}\widehat{\mathbf{g}}-\mathbf{X}\boldsymbol{\upbeta } -\mathbf{W}\widehat{\mathbf{v}}=\mathbf{e}$, the Eq. (16) can be written as:

17 $$ \begin{array}{l} logp\left(\mathbf{v}\Big|\mathbf{y}\right)= logf\left(\mathbf{y}\Big|\mathbf{v},\widehat{\mathbf{g}},\widehat{\boldsymbol{\upbeta}},\widehat{\sigma_e^2}\right)+ logp\left(\mathbf{v}\right)\\ {}=\left[-\frac{\mathrm{n}}{2} \log \widehat{\upsigma_{\mathrm{e}}^2}- \log \left|\mathbf{E}\right|+\frac{1}{2\widehat{\upsigma_{\mathrm{e}}^2}}\mathbf{e}\mathit{\hbox{'}}\;{\mathbf{E}}^{-1}\mathbf{e}\right]\\ {}+\left[-\frac{\mathrm{q}}{2}{ \log \upsigma}_{\mathrm{a}}^2- \log \left|\mathbf{A}\right|+\frac{1}{2{\upsigma}_{\mathrm{a}}^2}\mathbf{v}\mathit{\hbox{'}}\;{\mathbf{A}}^{-1}\mathbf{v}\right]\end{array} $$

Taking expectation over the missing data **e**, we get:

18 $$ \begin{array}{l}{\mathrm{E}}_{\mathbf{e}\Big|\mathbf{y}} logp\left(\mathbf{v}\Big|\mathbf{y}\right)=\left[-\frac{\mathrm{n}}{2} \log \widehat{\upsigma_{\mathrm{e}}^2}- \log \left|\mathbf{E}\right|+\frac{1}{2\widehat{\upsigma_{\mathrm{e}}^2}}\mathbf{e}\mathit{\hbox{'}}\;{\mathbf{E}}^{-1}\mathbf{e}+tr\left({\mathbf{E}}^{-1}\mathrm{P}\mathrm{E}\mathrm{V}\left(\mathbf{e}\right)\right)\right]\\ {}\kern9.5em +\left[-\frac{\mathrm{q}}{2}{ \log \upsigma}_{\mathrm{a}}^2- \log \left|\mathbf{A}\right|+\frac{1}{2{\upsigma}_{\mathrm{a}}^2}\mathbf{v}\mathit{\hbox{'}}\;{\mathbf{A}}^{-1}\mathbf{v}\right]\end{array} $$

Differentiating the Eq. (18) with regards to **v**, we get:

19 $$ \widehat{\mathbf{v}}={\left(\mathbf{W}\mathit{\hbox{'}}\;{\mathbf{E}}^{-1}\mathbf{W}{\upsigma}_{\mathrm{a}}^2+{\upsigma}_{\mathrm{e}}^2{\mathbf{A}}^{-1}\right)}^{-1}{\upsigma}_{\mathrm{a}}^2\mathbf{W}\hbox{'}{\mathbf{E}}^{-1}\left(\mathbf{y}-\mathbf{Z}\widehat{\mathbf{g}}-\mathbf{X}\widehat{\boldsymbol{\upbeta}}\right) $$

Where, for simplicity, the variance σ_a_^2^. will be fixed as the specified value from GBLUP estimation.

Table 1 lists all the parameters and their equation derived from EM steps.

**Table 1:** The list of all the estimated parameters including the possibility for each SNP (**P**(**i**, **k**)), the proportion parameter (**Pr**), each SNP effect (**g**_**i**_), error variance (**σ**_**e**_^2^), fixed effect (**β**), and polygenic effects **v** and the according equation derived from EM steps

Parameters	The data model	According equations derived from EM
${E}_{e^{*}} logP\left(i,k\right)$	The expected likelihood parameters for *P*(*i*, *k*)	Equation (S3)
*P*(*i*, *k*)	SNP effects related parameters under the extended model (3)	Equation (S4)
Pr		Equation (10)
*g*_*i*_		Equation (8)
σ_e_^2^	The overall model (1)	Equation (12)
β		Equation (13)
v		Equation (19)

#### Steps for EM module

The overall procedure of EM is described by means of the pseudo code, steps ①~⑦. Here we will detail these steps according to their sequence appearing in the pseudocode descriptions:

- Step *EM*_①: Initialise the parameters **g**, **Pr**, **σ**_**i**_^2^ and Construct **X, A, G, E, W** matrices. Similar to emBayesR [30], the starting values of **g** and **Pr** were set as **g** = 0.01 and Pr = {0.5, 0.487, 0.01, 0.003}, while **σ**_**i**_^2^ = {0, 0.0001 * σ_g_^2^, 0.001 * σ_g_^2^, 0.01 * σ_g_^2^}. The genetic variance σ_g_^2^ and polygenic variance σ_a_^2^ are obtained from GBLUP. Both variances won’t be updated during EM iterations.
  The *n* × 3 matrix **X** is a design matrix, allocating the phenotypes to fixed effects. In our case, the matrix **X** is set up with the first column being the mean, the second and third columns defining the breeds (Holstein or Jersey) and sex (bulls or cows) of the cattle. For example, if the *i*^*th*^ animal is a Holstein bull, then *x*_*i*,2_ = 1 with *x*_*i*,3_ = 0.
  The Pedigree relationship matrix **A** is built using Henderson’s rules [37]; while the genomic relationship matrix **G** is constructed using the equation **G** = **ZZ** ' /*n*. Diagonal error matrix **E** is calculated following Garrick et al. [36], and the index matrix **W** maps individuals in the pedigree to the phenotypes if they have ones.
- Step *EM*_②: Calculate the PEV matrix under model 1 (Additional file 1). Then using PEV matrix, calculate $tr\left({\mathbf{E}}^{-1}{\mathbf{Z}}_{\mathbf{i}}{\mathbf{Z}}_{\mathbf{i}}^{\mathit{\hbox{'}}}{\mathbf{E}}^{-1}\mathbf{P}\mathbf{E}{\mathbf{V}}_{{\mathbf{u}}_1}\left(\mathbf{e}\right)\right)$ which is used in the equation for ${E}_{e^{*}} logP\left(i,k\right)$ (Additional file 2). In theory, the calculation of this term should be updated each EM iteration, which is time consuming. To avoid huge computational burden, the PEV matrix is treated as constant value for the term $tr\left({\mathbf{E}}^{-1}{\mathbf{Z}}_{\mathbf{i}}{\mathbf{Z}}_{\mathbf{i}}^{\mathit{\hbox{'}}}{\mathbf{E}}^{-1}\mathbf{P}\mathbf{E}{\mathbf{V}}_{{\mathbf{u}}_1}\left(\mathbf{e}\right)\right)$ in front of EM loop.
  Then for each SNP *i* (*i* in 1 to *m*)
- Step *EM*_③: Correct **y** for the effects of all other SNPs except current SNP *i* with equation ${\mathbf{y}}^{\dagger }=\mathbf{y}-{\displaystyle {\sum}_{\mathrm{j}\ne \mathrm{i}}\kern0.5em {\mathbf{Z}}_{\mathbf{j}}}\kern0.5em {\widehat{\mathrm{g}}}_{\mathrm{j}}-\mathbf{X}\widehat{\mathbf{b}}-\mathbf{W}\widehat{\mathbf{v}}.$
- Step *EM*_④: Estimate the probability that the effect of SNP *i* is from one of four normal distributions ${E}_{e^{*}} logP\left(i,k\right)$ with the equation (S3). After this, *P*(*i*, *k*) is calculated with the equation $exp\left({E}_{e^{*}} log{P}_{ik}/{\displaystyle {\sum}_{k=1}^4 exp\kern0.5em }\left({E}_{e^{*}} log{P}_{ik}\right)\right)$ (S4).
- Step *EM*_⑤: the SNP effect ĝ_*i*_ is updated via Eq. (8).
  After effects have been estimated for all SNP,
- Step *EM*_⑥: Estimate *σ*_*e*_^2^ with Eq. (12), fixed effects **β** with Eq. (13), update *Pr*_*k*_ with Eq. (10), and update polygenic effects **v** with the Eq. (19).
- Step *EM*_⑦: Assess convergence criterion (**ĝ**^*l*^ − **ĝ**^*l* − 1^) ' (**ĝ**^*l*^ − **ĝ**^*q* − 1^)/((**ĝ**^*l*'^**ĝ**^*l*^) ≤ 10^− 10^ with *l* being the EM iterations number. If not converged, then return to Step ③ for the next EM iteration; otherwise, exit the EM iterations and return the estimates of parameters from the final iterations.

### Modified MCMC module with speed-up scheme

The outputs of the EM including SNP solutions, polygenic effects, error variance and genetic variance are used as starting values of parameters for the MCMC module, which allows MCMC to begin with no burn in.

The MCMC module of HyB_BR implements the same Gibbs sampling processes as BayesR [15] but modified with one speed-up scheme as follows. Over the first 500 iterations, the average probability that the SNP effect is zero (*p*(*i*, 1)) is calculated. If *p*(*i*, 1) ≥ a, then the SNP effect is set to zero and is not updated in future iterations.

**Fig. 1:**
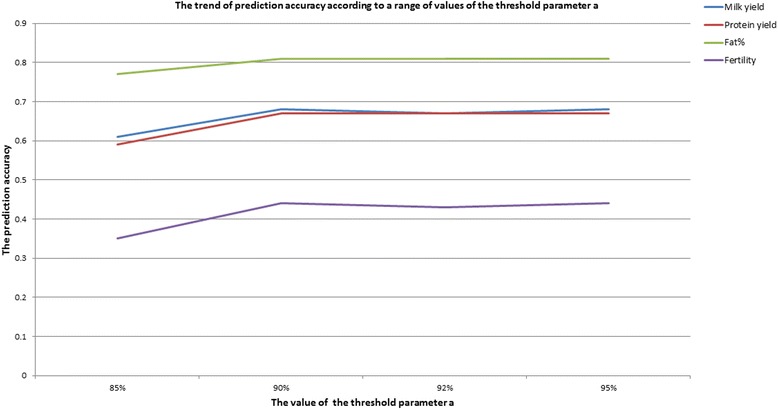
The trend of prediction accuracy according to a range of values of the threshold parameter **a**

The test for selecting a reasonable value of the parameter *a* was conducted as follows. The impact of value of *a* from 0.85 to 0.95 on prediction accuracy was investigated for the milk production traits and fertility, Fig. 1. The results show that criterion *p*(i, 1)≥, 1, is the lowest threshold which gives an accuracy very close to the maximum. The criterion means SNP *i* has more than 90 % probability of having no effect.

The modified MCMC steps can then be described as follows:

- Step *MCMC*_①: sampling the error variance ${\widehat{\upsigma}}_{\mathrm{e}}^2$ according to the distribution ${\widehat{\upsigma}}_{\mathrm{e}}^2\sim Inv-{\upchi}^2\left(n-2,\frac{{{\mathbf{y}}^{*}}^{\hbox{'}}{\mathbf{E}}^{-1}{\mathbf{y}}^{*}}{n-2}\right)$, with ${\mathbf{y}}^{*}=\left(\mathbf{y}-\mathbf{Zg}-\mathbf{X}\widehat{\boldsymbol{\upbeta}}-\mathbf{W}\widehat{\mathbf{v}}\right).$
- Step *MCMC*_②: sampling the fixed effects **β** from the distribution $N\Big(\ {\boldsymbol{\upbeta}}_{\mu },{\left(\mathbf{X}\mathit{\hbox{'}}\;{\mathbf{E}}^{-1}\mathbf{X}\right)}^{-1}{\widehat{\upsigma}}_{\mathrm{e}}^2$), with ${\boldsymbol{\upbeta}}_{\mu }={\left(\mathbf{X}\mathit{\hbox{'}}\;{\mathbf{E}}^{-1}\mathbf{X}\right)}^{-1}\mathbf{X}\mathit{\hbox{'}}\;{\mathbf{E}}^{-1}\left(\mathbf{y}-\mathbf{Z}\widehat{\mathbf{g}}-\mathbf{W}\widehat{\mathbf{v}}\right)$.
- Step *MCMC*_③: Polygenic variance is sampled ${\widehat{\upsigma}}_{\mathrm{a}}^2\sim Inv-{\upchi}^2\left(n-2,\frac{\widehat{\mathbf{v}}\mathit{\hbox{'}}\;{\mathbf{A}}^{-1}\widehat{\mathbf{v}}}{n-2}\right)$.
- Step *MCMC*_④: The polygenic effects are sampled from normal distribution *N*(*μ*, *s*), with the mean $\mu =\widehat{\mathbf{v}}$ from Eq. (19) and the variance$s={\left(\mathbf{W}\mathit{\hbox{'}}\;{\mathbf{E}}^{-1}\mathbf{W}+{\mathbf{A}}^{-1}{\upsigma}_{\mathrm{e}}^2/{\upsigma}_{\mathrm{a}}^2\right)}^{-1}.$
  Then for each SNP *i* (*i* in 1 to *m*),
- Step *MCMC*_⑤: Implement the speed-up scheme : if (iterations > 500) and (*P*(*i*, 1) > 0.9), then stop updating this SNP *i*.
  Else,
- Step *MCMC*_⑥: Estimate the log likelihood that the effect of SNP *i* is from one of four normal distributions *L*(*g*_*i*_|σ_*i*_^2^[*k*]). Following the derivation steps of Kemper et al. [15], the optimised equation of the log likelihood function is
  $$ \begin{array}{c}L\left({g}_i\Big|{\upsigma}_i^2\left[k\right]\right)=-\frac{1}{2}\left\{ \log \left({\upsigma}_i^2\left[k\right]{\mathbf{Z}}_{\mathbf{i}}^{\boldsymbol{\hbox{'}}}{\mathbf{Z}}_{\mathbf{i}}+{\upsigma}_{\mathrm{e}}^2\right)+{\left(\left({e}^{*}\right)\mathit{\hbox{'}}\;{\mathbf{E}}^{-1}{\mathbf{Z}}_{\mathbf{i}}\right)}^2{\upsigma}_i^2\left[k\right]{\upsigma}_{\mathrm{e}}^{-2}/\left({\upsigma}_i^2\left[k\right]{\mathbf{Z}}_{\mathbf{i}}^{\boldsymbol{\hbox{'}}}{\mathbf{E}}^{-1}{\mathbf{Z}}_{\mathbf{i}}+{\upsigma}_{\mathrm{e}}^2\right)\right\}\\ {}+ log{ \Pr}_{\mathrm{k}},\end{array} $$
  with e* = **y** − X**β** − **u** − **Wv**.
  After this, *P*(*i*, *k*) is calculated with the equation:$exp\Big(L\left({g}_i\Big|{\upsigma}_i^2\left[k\right]\right)/{\displaystyle {\sum}_{k=1}^4 exp\kern0.5em }\left(L\left({g}_i\Big|{\upsigma}_i^2\left[k\right]\right)\right)$
- Step *MCMC*_⑦: Sample ĝ_*i*_ with N(*μ*, *s*), $\mu ={\left[{\mathbf{Z}}_{\mathrm{i}}^{\mathit{\hbox{'}}}{\mathbf{E}}^{-1}{\mathbf{Z}}_{\mathrm{i}}+\frac{\widehat{\upsigma_{\mathrm{e}}^2}}{\upsigma_i^2\left[k\right]}\right]}^{-1}\left[{\mathbf{Z}}^{\mathit{\hbox{'}}}{\mathbf{E}}^{-1}\ {e}^{*}\right]$, and $s={\left[{\mathbf{Z}}_{\mathrm{i}}^{\mathit{\hbox{'}}}{\mathbf{E}}^{-1}{\mathbf{Z}}_{\mathrm{i}}+\frac{\widehat{\upsigma_{\mathrm{e}}^2}}{\upsigma_i^2\left[k\right]}\right]}^{-1}$.
- Step *MCMC*_⑧: Update Pr ~ Dirichlet(β_1_ + 1, β_2_ + 1, β_3_ + 1, β_4_ + 1),where β_1_, β_2_, …, β_4_ are the number of SNPs in one of four normal distributions.
  Return to MCMC step 1.
  HyB_BR was written in the C++ programming language.

### Data

#### Cattle

One thousand seven hundred forty-five Holstein and Jersey cattle and 114 Australian Red bulls were genotyped with the 777 K Illumina HD bovine SNP chip. 15,049 Holstein and Jersey bulls and cows, 249 Australian red bulls and cows were genotyped with the 54 K Illumina Bovine SNP array. After stringent quality control and SNP filtering described in [14], there were 632,003 SNPs remaining for animals genotyped with the 777 K SNP panel, and 43,425 SNPs remaining for animals genotyped with the 54 K SNP array. Animals genotyped with the 43,425 SNPs, were imputed to 632,003 SNP genotypes using Beagle 3.0 [38]. Therefore, the total data set was 17,157 cattle of three breeds with real or imputed genotypes for 632,003 SNP.

The phenotypes include milk yield, protein yield, fat percent(fat%), and cow fertility. The heritability of these traits varies from 0.33 (for milk yield, protein yield and fat%), to 0.03 (for cow fertility). The fertility (reproductive performance of dairy cows) is usually measured according to calving interval (CI, the number of days between successive calvings), days from calving to first service (CFS), pregnancy diagnosis, lactation length (LL), and survival to second lactation on Australian Holstein and Jersey cows [39, 40]. Here, the fertility phenotype was calving interval (CI). Here, the fertility phenotype is mainly derived from CI. The phenotypes for all the traits were daughter trait deviations (DTD) for bulls (the average of their daughters phenotypes, corrected for fixed effects), and trait deviations (TD) for cows (as described by Kemper et al. [15]). For genomic prediction, the data was separated into a reference set, where SNP effects were estimated, and validation sets, where the prediction accuracy was assessed, and the division of animals into reference and validation sets was by year of birth (youngest animals in validation set). The reference data includes bulls and cows from two breeds (Holstein and Jersey), and the predictions were evaluated in the other animals of the same breeds or in a breed (Aussie red) not included in the reference set. The exact number of individuals in these data sets for each trait is given in Table 2.

**Table 2:** The number of individuals in the reference sets and validations sets related to three traits including Milk yield (MilkY), Protein yield (ProtY), Fat Percent (Fat%) and Fertility

Traits	Reference Sets				Validation Sets		
	Holstein		Jersey		Holstein Bulls	Jersey Bulls	Australian Red Bulls
	Bulls	Cows	Bulls	Cows			
MilkY/ProtY/Fat%	3049	8478	770	3917	262	105	114
Fertility	2806	7838	716	3830	396	81	114

To compare the computational time required by the different genomic prediction methods, we also used three reference sets with increasing different numbers of animals; Ref 1_ CATTLE had 3049 Holstein bulls; Ref 2_CATTLE had 11,527 Holstein bulls and cows, while Ref 3_CATTLE was the complete reference data set with 16,214 animals.

For the EM module, estimates of three variance components (*σ*_*e*_^2^, *σ*_*v*_^2^, *σ*_*g*_^2^) were required as the input. We ran Asreml4.0 [41] (which is implemented with GBLUP methods) on these data sets to estimate these variance parameters and the results are listed in Table 3.

**Table 3:** Three input variance parameters related to the reference data sets

Reference Set	Traits	σ_e_^2^	σ_g_^2^	σ_v_^2^
Holstein and Jersey bulls & cows	Milk yield	133284.0	108619.0	34925.6
	Protein yield	132.579	68.6635	29.1662
	Fat%	0.0180012	0.0575729	0.0127094
	Fertility	3283.80	31.6187	0.000332297

The variances including error variance (**σ**_**e**_^2^), genetic variance (**σ**_**g**_^2^), and polygenic variance (**σ**_**v**_^2^) are estimated by ASReml 4

The correlation between GEBV and DTD in the validation sets was used as a proxy for prediction accuracy. The regression of DTD on GEBV in the validation sets was used to investigate if any of the methods resulted in biased predictions.

#### Case/Control human disease trait data

For predicting human disease risk, seven disease traits of the Welcome Trust Case Control Consortium (WTCCC) genomic data [27] including bipolar disorder (BD), coronary artery disease (CAD), Crohn’s disease (CD), Hypertension (HT), rheumatoid arthritis (RA), type 1 diabetes (T1D), and type 2 diabetes (T2D) were used. Following the steps of strict QC on SNP data [7, 8, 42] with Plink [43], we had seven combined case/control data sets (one for each trait) with different number of markers and records listed in Table 4. The input parameters of seven datasets for HyB_BR including error variance and genetic variance were calculated by GCTA [44], given in Table 4. To assess prediction accuracy, for each disease, we randomly generated 20 splits of the data with 80 % of individuals for the reference set and 20 % for the validation set. To assess the prediction ability, the area under the ROC curve (AUC) [45] was calculated.

**Table 4:** The size and genetic architecture of seven combined control/case data sets

Diseases	Number of records	Number of markers	σ_e_^2^	σ_g_^2^	*h* ^2^
BD	4722	292,496	0.070509	0.17156	0.71
CAD	4864	296,610	0.149782	0.09189	0.38
CD	4577	301,579	0.073900	0.16056	0.69
HT	4890	294,404	0.113621	0.12816	0.53
RA	4704	295,890	0.070900	0.07120	0.50
T1D	4824	296,228	0.064739	0.12567	0.66
T2D	4722	294,641	0.099866	0.14497	0.59

The error variance (σ_e_^2^) and genetic variance (σ_g_^2^) are estimated by GCTA; the heritability (*h* ^2^) is estimated by the equation $$ {h}^2=\raisebox{1ex}{${\upsigma}_{\mathrm{g}}^2$}\!\left/ \!\raisebox{-1ex}{$\left({\upsigma}_{\mathrm{e}}^2+{\upsigma}_{\mathrm{g}}^2\right)$}\right. $$

## Results

### Compute time comparison of HyB_BR and BayesR

**Fig. 2:**
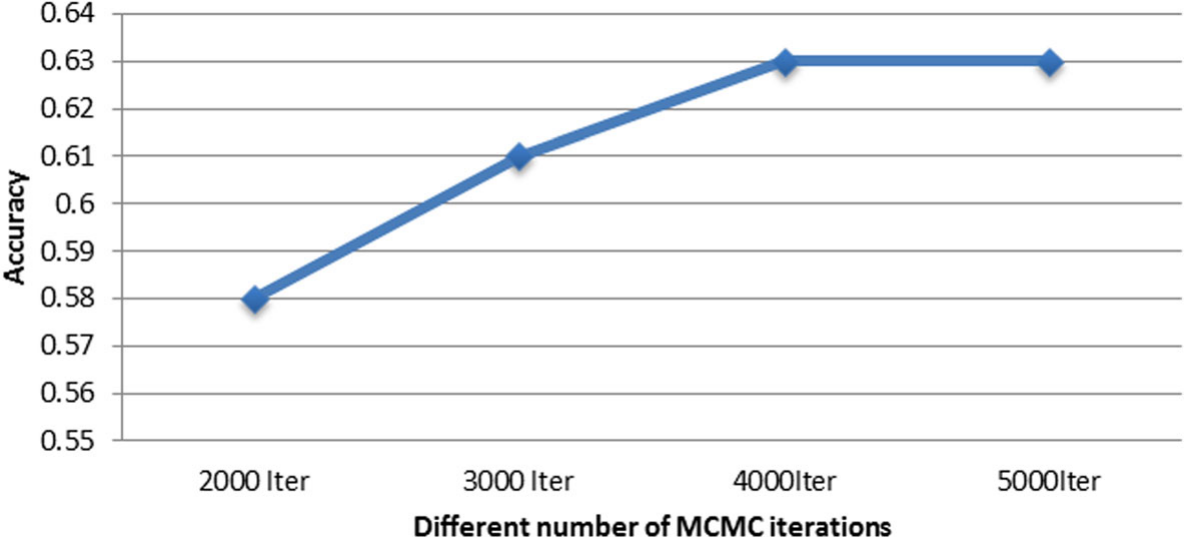
Prediction accuracy with an increasing number of MCMC iterations for BayesR

To compare computational efficiency, HyB_BR without the speed-up scheme (labelled as Hyb_BR_Orig), HyB_BR with the speed-up scheme and pure MCMC BayesR were implemented on three data sets with 632,003 markers but different numbers of records, varying from 3049 in Ref 1_CATTLE, 11,527 in Ref 2_CATTLE, to 16,214 in Ref 3_CATTLE. As used by Kemper et al. [15], pure MCMC BayesR required 40,000 iterations of complexity *O*(*mn*) with parameters estimated from samples from the posterior distributions (*m* is the number of markers and *n* is the number of individuals). The first 20,000 iterations were removed as burn in. The MCMC module of HyB_BR used only 4000 iterations and burn-in was replaced by the EM (400 iterations to convergence). 4000 cycles for the MCMC module were used after comparing results with increasing number of iterations. The results showed that 4000 were necessary to achieve maximum prediction accuracy (Fig. 2).

The prediction accuracy was evaluated for milk yield with a reference set containing the Holstein and Jersey bulls&cows data.

**Fig. 3:**
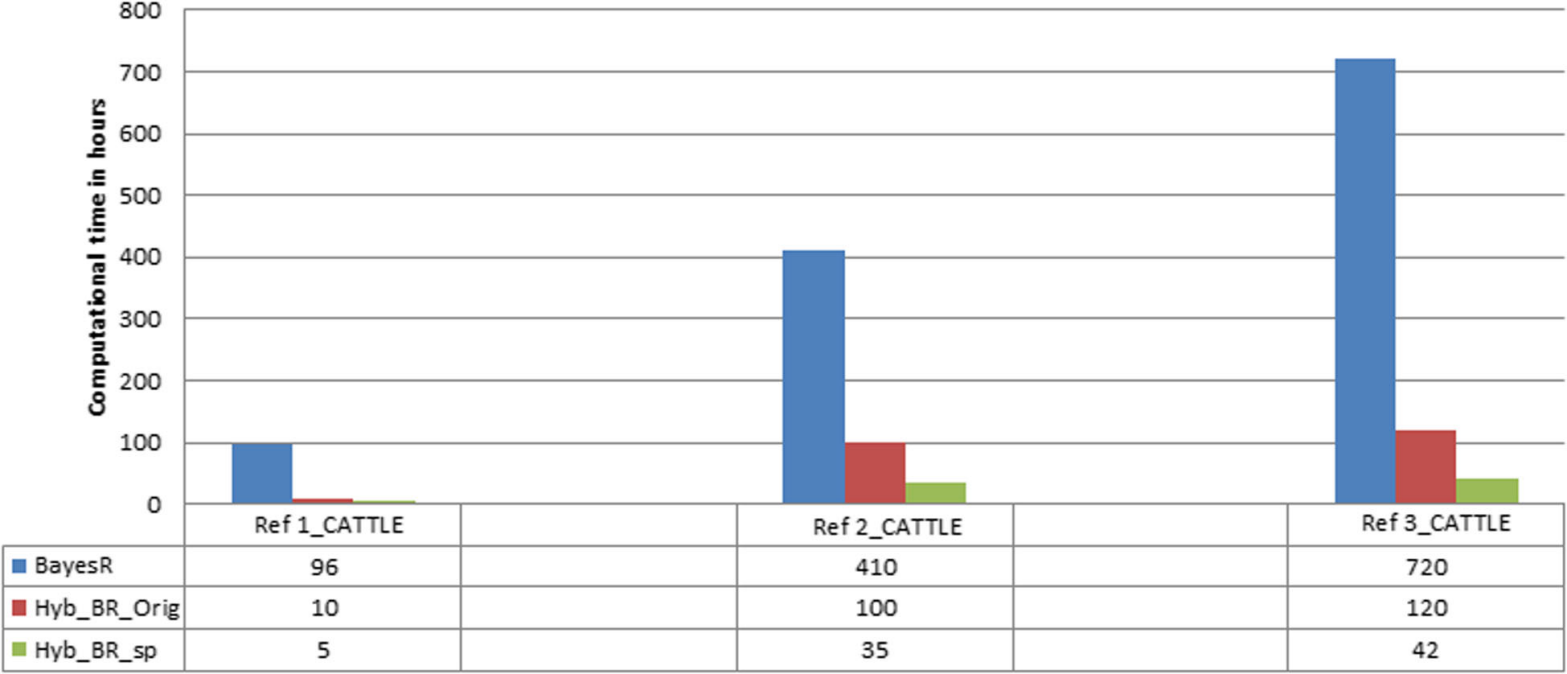
Computational time in hours required for BayesR, HyB_BR_Orig, and HyB_BR_sp on three reference sets (Ref 1_CATTLE, Ref 2_CATTLE, Ref 3_CATTLE)

With the smallest data set (Ref 1_CATTLE), 5 h compute time was required for HyB_BR compared with 96 h for BayesR MCMC (Fig. 3); 35 h required by HyB_BR instead of 410 h of BayesR for Ref 2_CATTLE; And in Ref 3_CATTLE, 42 h for HyB_BR_sp but 720 h for BayesR. These results suggest HyB_BR is at least 10 times faster than BayesR MCMC, with the speed advantage increasing as data sets became larger (17 times faster with the largest data set). The HyB_BR speed up scheme reduced compute time by approximately 50 %, compared to HyB_BR_Orig without the speed up scheme (Fig. 3), with no reduction in the prediction accuracy (Tables 5, 6 and 7).

**Table 5:** The accuracy and bias of with-breed prediction of GBLUP, BayesR(BR), emBayesR (EM), and HyB_BR (HB)

		Milk yield		Protein yield		Fat%		Fertility	
		Acc.	Bias	Acc.	Bias	Acc.	Bias	Acc.	Bias
		Holstein reference to predict Holstein validation							
GBLUP	+Poly^a^	0.57	0.96	0.63	0.98	0.73	0.96	0.43	1.26
	-Poly^b^	0.56	0.86	0.59	0.87	0.71	1.15	0.42	1.27
BR	+Poly^a^	0.63	0.91	0.64	1.01	0.79	1.06	0.43	1.19
	-Poly^b^	0.61	1.00	0.63	1.06	0.77	1.13	0.41	1.19
EM	+Poly^a^	0.62	0.79	0.63	0.85	0.77	0.98	0.42	1.15
	-Poly^b^	0.62	0.92	0.62	0.94	0.74	1.06	0.41	1.15
HB	+Poly^a^	0.63	0.93	0.63	0.97	0.79	1.09	0.43	1.19
	-Poly^b^	0.63	1.03	0.62	1.06	0.76	1.17	0.42	1.19
		Jersey reference to predict Jersey validation							
GBLUP	+Poly^a^	0.59	0.93	0.65	0.91	0.54	0.71	0.15	1.05
	-Poly^b^	0.58	1.05	0.64	1.09	0.54	0.77	0.14	1.08
BR	+Poly^a^	0.64	0.94	0.68	0.93	0.71	0.87	0.15	1.02
	-Poly^b^	0.63	0.98	0.68	1.01	0.69	0.93	0.14	1.04
EM	+Poly^a^	0.64	0.87	0.68	0.92	0.69	0.75	0.15	1.09
	-Poly^b^	0.64	0.98	0.66	1.01	0.67	0.79	0.15	1.09
HB	+Poly^a^	0.64	0.97	0.68	0.90	0.71	0.89	0.15	1.02
	-Poly^b^	0.64	1.06	0.66	0.96	0.69	0.87	0.15	1.02

^a^means polygenic effects are included in the predictions; while ^b^means the predictions do not include polygenic effects into the model

**Table 6:** The accuracy and bias of multi-breeds prediction of GBLUP, BayesR(BR), emBayesR (EM), and HyB_BR (HB)

		Milk yield		Protein yield		Fat%		Fertility	
		Acc.	Bias	Acc.	Bias	Acc.	Bias	Acc.	Bias
		Holstein and Jersey reference to predict Holstein validation							
GBLUP	+Poly^a^	0.63	0.83	0.65	0.85	0.74	0.85	0.44	1.66
	-Poly^b^	0.62	0.90	0.57	0.88	0.72	0.90	0.42	1.66
BR	+Poly^a^	0.68	0.84	0.68	0.88	0.81	0.90	0.44	1.53
	-Poly^b^	0.67	0.91	0.67	1.03	0.79	0.98	0.42	1.53
EM	+Poly^a^	0.68	0.90	0.68	0.79	0.77	0.83	0.44	1.27
	-Poly^b^	0.65	0.91	0.66	0.85	0.75	0.87	0.44	1.27
HB	+Poly^a^	0.68	0.82	0.67	0.88	0.81	0.94	0.44	1.33
	-Poly^b^	0.67	0.89	0.67	0.95	0.80	1.08	0.44	1.33
		Holstein and Jersey reference to predict Jersey validation							
GBLUP	+Poly^a^	0.64	0.78	0.68	0.85	0.66	0.73	0.24	1.12
	-Poly^b^	0.64	0.90	0.69	1.02	0.64	0.80	0.24	1.12
BR	+Poly^a^	0.69	0.85	0.71	0.99	0.76	0.88	0.26	1.23
	-Poly^b^	0.68	0.95	0.71	1.09	0.74	0.94	0.25	1.24
EM	+Poly^a^	0.66	0.84	0.69	0.71	0.75	0.76	0.23	1.13
	-Poly^b^	0.63	0.86	0.68	0.73	0.70	0.82	0.23	1.13
HB	+Poly^a^	0.71	0.89	0.74	0.94	0.77	0.89	0.26	1.02
	-Poly^b^	0.69	0.98	0.73	1.02	0.73	0.97	0.26	1.02

^a^means polygenic effects are included in the predictions; while ^b^means the predictions do not include polygenic effects into the model

**Table 7:** The accuracy and bias of across-breeds prediction of BayesR, GBLUP, and HyB_BR

	Milk yield		Protein yield		Fat%		Fertility	
	Acc.	Bias	Acc.	Bias	Acc.	Bias	Acc.	Bias
	Across breeds prediction on Australian red bulls							
GBLUP	0.16	0.54	0.11	0.51	0.32	0.90	0.29	0.97
BayesR	0.22	0.60	0.12	0.49	0.45	0.92	0.27	1.03
EmBayesR	0.24	0.70	0.12	0.42	0.41	0.89	0.29	1.10
HyB_BR	0.23	0.74	0.17	0.49	0.50	0.90	0.30	0.98
	Across breeds prediction on Australian red cows							
GBLUP	0.15	0.71	0.08	0.13	0.50	1.19	0.08	0.79
BayesR	0.26	0.80	0.17	0.51	0.54	0.94	0.08	0.68
EmBayesR	0.24	0.79	0.16	0.53	0.51	0.89	0.08	0.74
HyB_BR	0.26	0.81	0.16	0.57	0.55	0.91	0.08	0.70

These timings were recorded on a server with Intel E5-2680 2.7GHz processors and 384GB of 1333 MHz RAM.

### The accuracy and bias of within-breeds, multi-breeds and across-breeds prediction for four complex dairy traits

#### Genomic prediction with a single breed reference

For the within-breed prediction (that is, when a Holstein reference was used to estimate SNP effects used for calculating GEBV in a Holstein validation set, and likewise for Jersey) in Table 5, HyB_BR performed as well as BayesR for all traits, including fat%. Both BayesR and HyB_BR had a 1 % ~ 6 % superiority of accuracy over GBLUP for Milk yield, Protein yield and Fat%, but had no advantage for fertility. Similarly, for the prediction of Jersey validation with Jersey reference, BayesR and HyB_BR had a consistent advantage over GBLUP for milk production traits, but not for fertility. Especially, for the trait Fat%, BayesR and HyB_BR gave very similar results, with a 17 % increase in accuracy (0.79 vs 0.73 in Holstein and 0.71 vs 0.54 in Jersey) of genomic prediction over GBLUP, as well as a 5 % increase in accuracy over emBayesR. HyB_BR and BayesR also gave regression coefficients closer to one than GBLUP for most traits.

#### Genomic prediction with a multi-breed reference

When predicting the Holstein or Jersey validation with the combined Holstein and Jersey reference, HyB_BR had the same accuracy as BayesR, Table 5. Compared with GBLUP, BayesR and HyB_BR gave consistently higher accuracy increase for the milk production traits, though this was not observed for fertility. And for the prediction of Jersey validation set, BayesR and HyB_BR improved accuracy for the milk production traits by 11 % compared with GBLUP. The results show that there are small but consistent accuracy improvements as a result of using the multi-breed reference (compare Tables 5 and 6), consistent with the results of Kemper et al. [15] and Hoze et al. [46].

Also, including polygenic effects (estimated using the pedigree) in the model can improve the prediction accuracy by 1 ~ 2 %, at least for milk production traits, Tables 5 and 6. However, for fertility the introduction of polygenic effects for all the prediction methods did not impact the accuracy at all.

Compared with GBLUP and emBayesR, BayesR and HyB_BR gave less biased predictions for milk production traits. However for fertility the regression values far from one indicate bias, from all methods – the low accuracy of fertility phenotypes, including in the validation set, likely contributes to this.

#### Genomic prediction across breeds

For predicting Australian Red validation bulls (an additional breed to those in the reference set), BayesR and HyB_BR gave higher accuracy than GBLUP for all traits (Table 7).

Across all the prediction results shown in Tables 5, 6 and 7, emBayesR had a 2 % ~ 5 % reduction in accuracy compared with BayesR and HyB_BR for fat%, while BayesR and HyB_BR gave almost identical accuracies in all cases.

### Inferred genetic architecture and QTL mapping for dairy production and fertility traits

Bayes R described the genetic architecture of a trait by the posterior proportion of SNPs in each of the four different distributions. Table 8 reported the estimated proportion in each of four distributions from BayesR, emBayesR, and HyB_BR. The number of SNPs falling into the distribution with the largest variance was similar for all three methods. Compared with BayesR, HyB_BR tended to estimate more SNPs (up to 5 %) in the distribution with variance 0.001 * *σ*_*g*_^2^, and 0.0001 * *σ*_*g*_^2^. In contrast to HyB_BR, emBayesR tended to estimate that a higher proportion of SNPs have no effect than does BayesR. This may explain the lower accuracy it achieves.

**Table 8:** The number of SNPs in each of four distributions

Traits	The proportion (Pr)	BayesR	emBayesR	HyB_BR
Milk yield	A. 0.01 * *σ*_*g*_^2^	8	6	8
	B. 0.001 * *σ*_*g*_^2^	47	17	327
	C. 0.0001 * *σ*_*g*_^2^	3886	1523	4039
	D. 0	628,062	630,457	627,629
Protein yield	A. 0.01 * *σ*_*g*_^2^	5	4	6
	B. 0.001 * *σ*_*g*_^2^	32	37	297
	C. 0.0001 * *σ*_*g*_^2^	4431	1842	6604
	D. 0	627,535	630,120	625,096
Fat%	A. 0.01 * *σ*_*g*_^2^	23	19	20
	B. 0.001 * *σ*_*g*_^2^	46	206	119
	C. 0.0001 * *σ*_*g*_^2^	2882	1206	1852
	D. 0	629,052	630,572	630,012
Fertility	A. 0.01 * *σ*_*g*_^2^.	10	8	12
	B. 0.001 * *σ*_*g*_^2^	147	114	202
	C. 0.0001 * *σ*_*g*_^2^	3949	8572	7597
	D. 0	627,897	623,309	624,192

### QTL mapping for dairy production and fertility traits

Both BayesR and HyB_BR estimate the posterior probability that every SNP has a non-zero effect on the trait. This is useful for QTL mapping – SNP with very high posterior probabilities of having a non-zero effect should be strongly associated with causal mutations (e.g. Moser et al. [8], Kemper et al. [15]). Then, QTL mapping from BayesR and HyB_BR can be conducted by plotting the posterior probability of each SNPs having a non-zero effect on the trait by genome position, and then comparing the genome location of the effects with a high posterior probability of being in the largest distribution for each method.

**Fig. 4:**
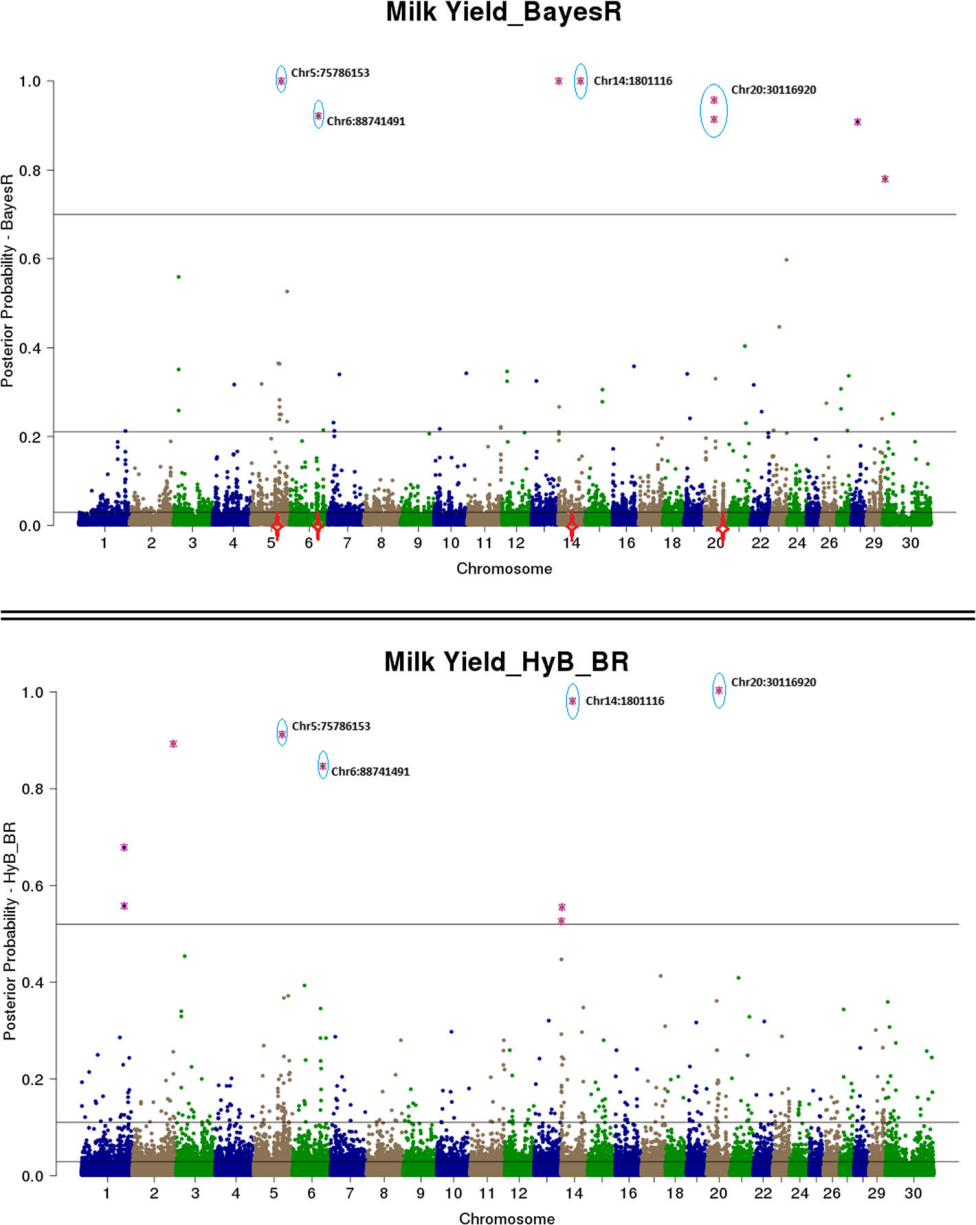
Mapping all the SNPs’ posterior possibilities estimated from BayesR and HyB_BR across the whole chromosome related to milk yield. The posterior possibility is calculated based on the sum of the posterior possibilities *P*(*i*, *k*) of each SNP with non-zero variances written as ∑_*k* = 2_^4^ *P*(*i*, *k*). The *blue circle* is the SNPs (picked up based on the high posterior possibility following in the distribution with largest variances) with location information mapped to known genes

**Fig. 5:**
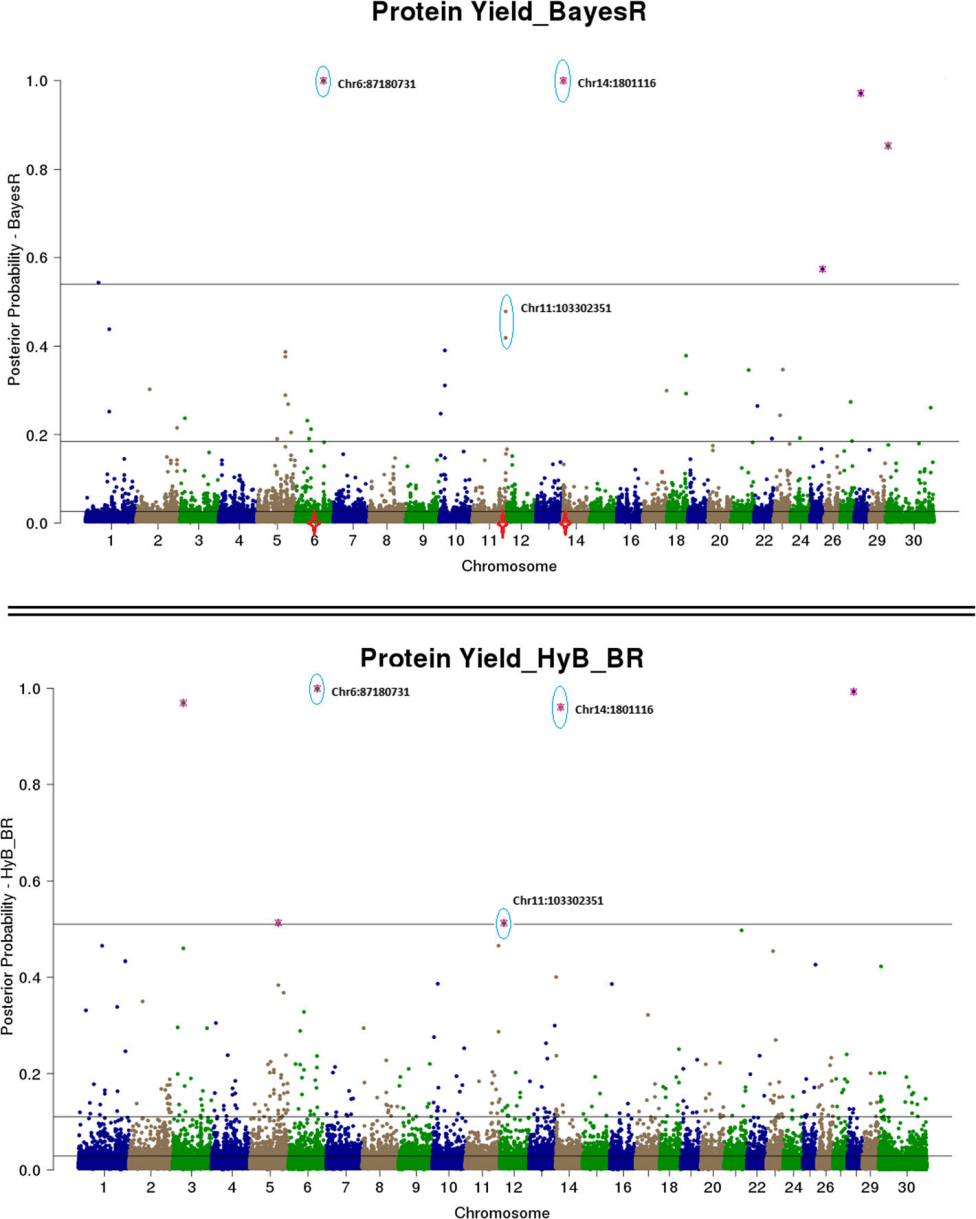
Mapping all the SNPs’ posterior possibilities estimated from BayesR and HyB_BR across the whole chromosome related to protein yield. The posterior possibility is calculated based on the sum of the posterior possibilities *P*(*i*, *k*) of each SNP with non-zero variances written as ∑_*k* = 2_^4^ *P*(*i*, *k*). The *blue circle* is the SNPs (picked up based on the high posterior possibility following in the distribution with largest variances) with location information mapped to known genes

**Fig. 6:**
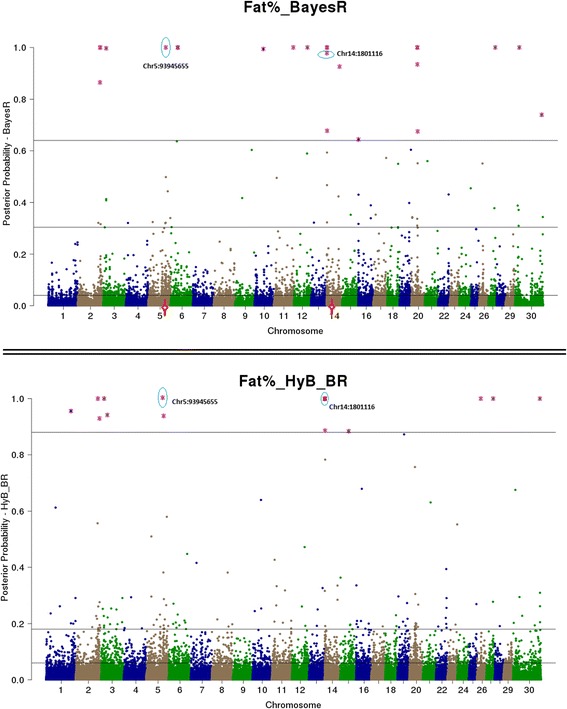
Mapping all the SNPs’ posterior possibilities estimated from BayesR and HyB_BR across the whole chromosome related to Fat percent (Fat%). The posterior possibility is calculated based on the sum of the posterior possibilities *P*(*i*, *k*) of each SNP with non-zero variances written as ∑_*k* = 2_^4^ *P*(*i*, *k*). The *blue circle* is the SNPs (picked up based on the high posterior possibility following in the distribution with largest variances) with location information mapped to known genes

**Fig. 7:**
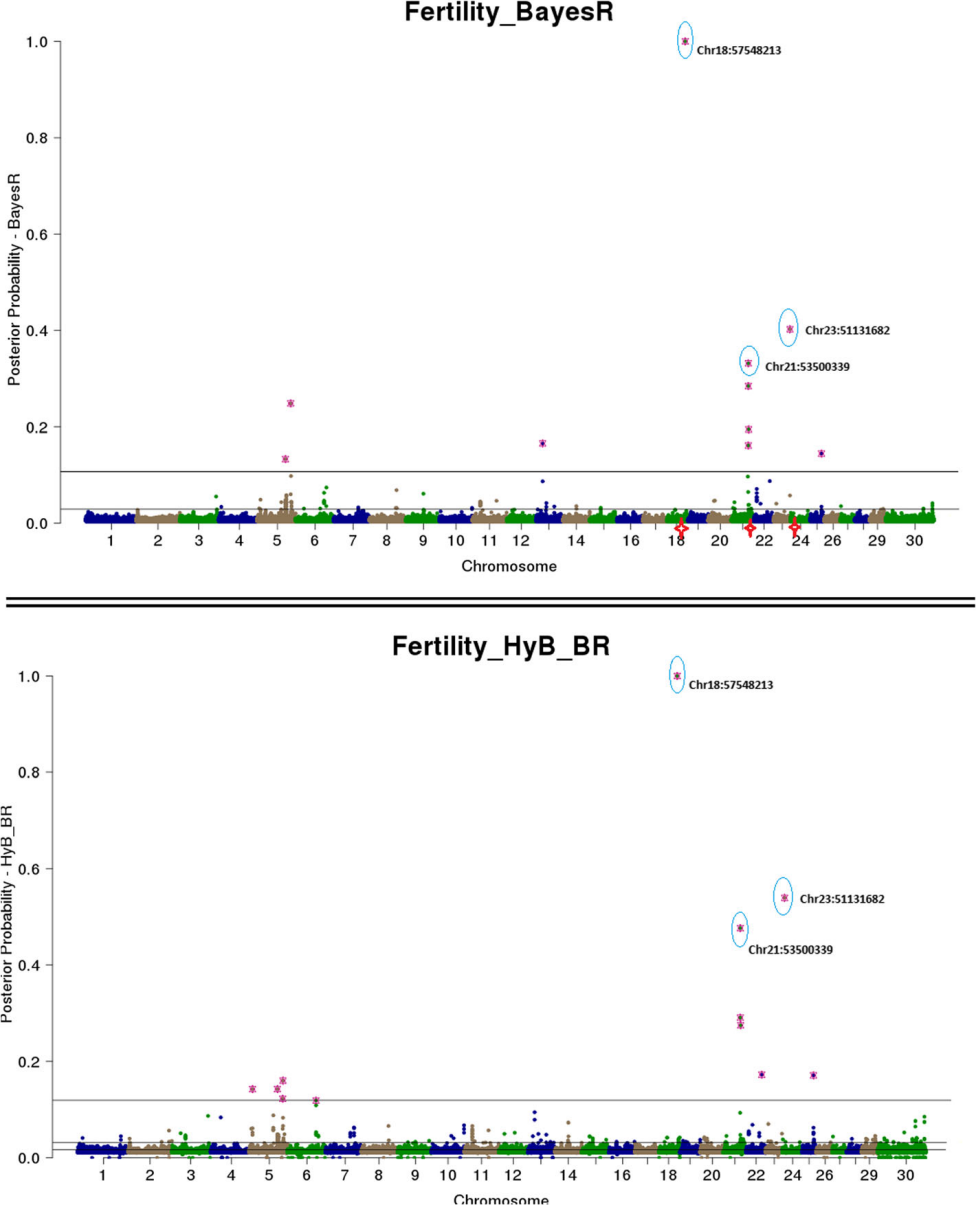
Mapping all the SNPs’ posterior possibilities estimated from BayesR and HyB_BR across the whole chromosome related to fertility. The posterior possibility is calculated based on the sum of the posterior possibilities *P*(*i*, *k*) of each SNP with non-zero variances written as ∑_*k* = 2_^4^ *P*(*i*, *k*). The *blue circle* is the SNPs (picked up based on the high posterior possibility following in the distribution with largest variances) with location information mapped to known genes

The estimated posterior possibilities of all the SNPs (y axis) related to four different traits were plotted according to the positions (base pairs) of SNPs on the whole genome (x axis) in Figs. 4, 5, 6 and 7. The top SNPs with high posterior possibilities were picked up according to the number of SNPs in the variance 0.01 * *σ*_*g*_^2^ (the total number of markers * Pr [4]). Table 9 listed all the top SNPs in the variance related to the previously reported genes with a effect on milk production including CSF2RB [47], GC [48], GHR/CCL28 [18], PAEP [17], MGST1 [49], and DGAT1 [16]. Both BayesR and HyB_BR identified all of these regions, which demonstrated that HyB_BR can perform QTL mapping with similar precision to BayesR. For example, HyB_BR could detect the DGAT1 as well as BayesR shown in Fig. 6 (Fat%).

**Table 9:** The list of identified causal mutations by both BayesR and HyB_BR

Traits	Loci	Information (known genes)	Range (bp) [Start points ~ End points]
Milk yield	Chr5:75786153	CSF2RB impacting milk yield [47].	[75724620 ~ 75745819]
	Chr6:88741491	GC, encoding the vitamin D binding protein, positively impacting the milk yield [48].	[88695940 ~ 88749180]
	Chr20:30116920	In association with CCL28/GHR impacting milk production [18].	[31890736 ~ 32199996]
Protein yield	Chr6:87180731	CSN1S1 positively impacting protein yield [48].	[87141556 ~ 87159096]
	Chr11:103302351	PAEP impacting protein yield [19].	[103301664 ~ 103306381]
Fat%	Chr5:93945655	MGST1 for Fat percent [49].	[93926791 ~ 3950162]
Fertility	Chr18:57548213	-In association with the gene CEACAM18, Detected by Pryce et al. [50], Cole et al. [51].	~57MBP
	Chr21:53500339	- Control the percentage of unassisted births in first calf heifers [52].	~53MBP
	Chr23:51131682	In the linkage with the known gene GMDS [53].	~51MBP
All the traits	Chr14:1801116	DGAT1 impacting Fat percent [16].	[1795351 ~ 1804562]

### The application of HyB_BR to predict the risk of Human disease traits and infer genetic architecture for these traits

In the human data, cross validation was used to estimate the accuracy of HyB_BR. As there were 20 replicates of 20/80 split (validation/reference), we evaluated the mean of the AUC for each disease shown in Table 10. Analysis methods compared were GBLUP implemented in GCTA [44], BayesR from Moser et al. [8], and HyB_BR. The standard deviations of the accuracy (across the 20 replicates) were also listed in the parenthesis of Table 10. HyB_BR and BayesR performed equally well across all seven traits, with the same prediction accuracy for each trait. For the diseases of CD, RA, and T1D, BayesR and HyB_BR had significantly higher accuracy than GBLUP. Especially for T1D, BayesR and HyB_BR could have up to 12 % accuracy increase compared with GBLUP. However, for other traits including BD, CAD, HT, and T2D, BayesR and HyB_BR did not show any superiority over GBLUP. The underlying architecture of these traits might explained this, as suggested by Moser et al. [8]. In detail, for CD, RA and T1D, there are known mutations of moderate to large effect, and the mixture assumptions of BayesR and HyB_BR can take advantage of this. However, for four other diseases including BD, CAD, HT, and T2D, there are no known mutations of moderate to large effect, and this is reflected in the genetic architecture for these diseases inferred by HyB_BR.

**Table 10:** The prediction performance evaluated by the Area under curve (AUC) of GBLUP, BayesR and HyB_BR on seven diseases

Diseases	GBLUP		BayesR		HyB_BR	
	AUC	*h* ^2^	AUC	*h* ^2^	AUC	*h* ^2^
BD	0.63(0.0135)	0.71	0.63(0.0131)	0.63	0.64(0.0174)	0.63
CAD	0.58(0.0116)	0.38	0.59(0.0118)	0.38	0.58(0.0131)	0.38
CD	0.60(0.0134)	0.69	0.65(0.0159)	0.61	0.65(0.0158)	0.61
HT	0.58(0.0125)	0.53	0.58(0.0131)	0.52	0.58(0.0140)	0.51
RA	0.58(0.0109)	0.50	0.70(0.0104)	0.45	0.70(0.0107)	0.45
T1D	0.64(0.0133)	0.66	0.86(0.0099)	0.63	0.86(0.0102)	0.63
T2D	0.59(0.0139)	0.59	0.60(0.0117)	0.52	0.60(0.0122)	0.52

the heritability (*h* ^2^) is estimated by the equation $$ {h}^2=\raisebox{1ex}{${\upsigma}_{\mathrm{g}}^2$}\!\left/ \!\raisebox{-1ex}{$\left({\upsigma}_{\mathrm{e}}^2+{\upsigma}_{\mathrm{g}}^2\right)$}\right. $$; σ_e_^2^ is derived separately by three methods; fixed genetic variance of σ_g_^2^ for BayesR and HyB_BR is obtained from GCTA

#### The genetic architecture of human disease traits

**Fig. 8:**
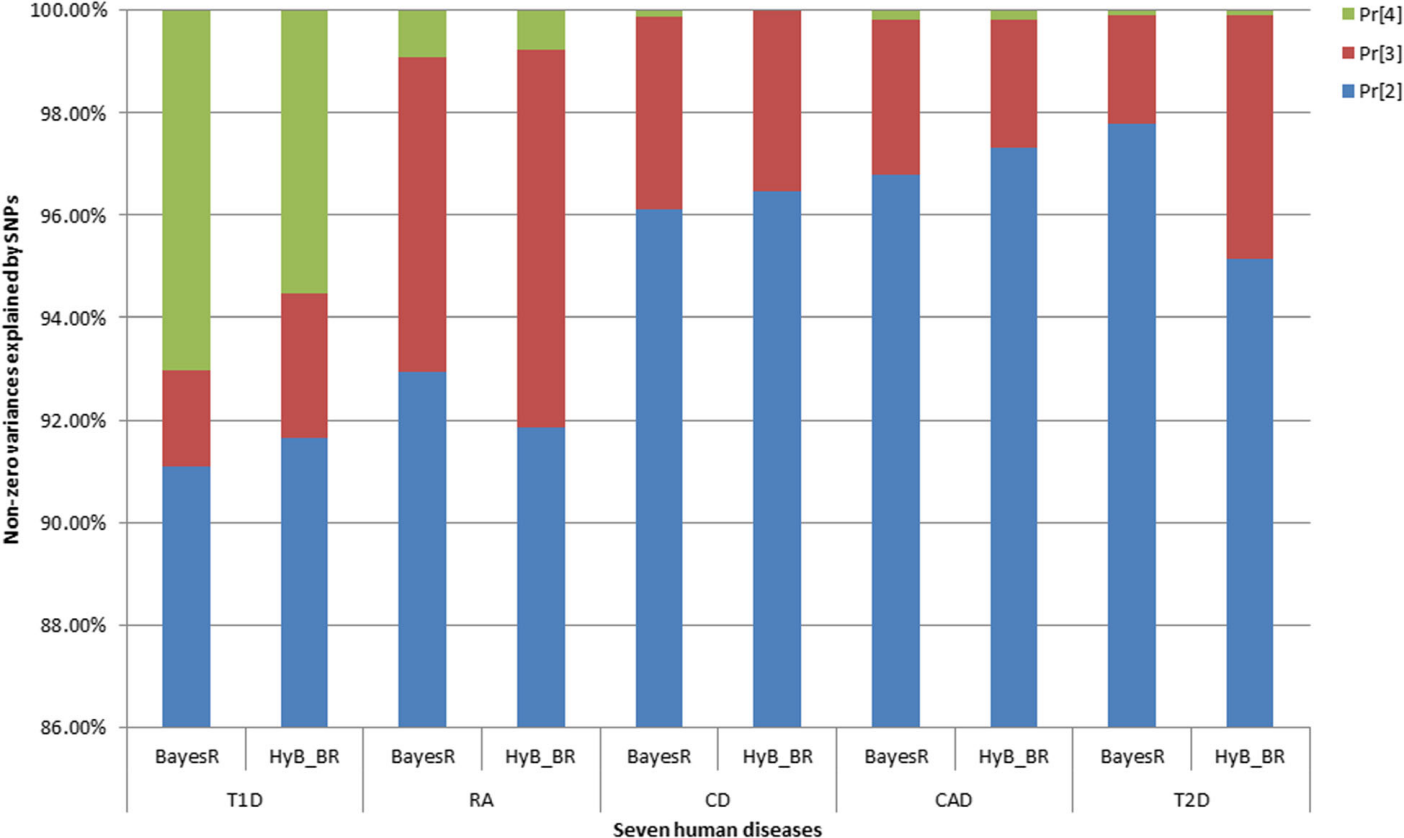
The inferred genetic architecture of seven human diseases from BayesR and HyB_BR. The *blue bar* is the proportion of SNPs in Pr [2] (with the variance 0.0001 * *σ*_*g*_^2^), which is estimated by the number of SNP in Pr [2] divided by the total number of SNPs with nonzero variance. The *red bar* is the proportion of SNPs with the variance 0.001 * *σ*_*g*_^2^, estimated by the number of SNP in Pr [3] divided by the total number of SNPs with nonzero variance. The *green bar* is the proportion of SNPs with the variances 0.01 * *σ*_*g*_^2^, estimated by the number of SNPs in Pr [2] divided by the total number of SNPs with nonzero variance

The inferred genetic architecture was different for each of the seven diseases (Table 11). For example, the genetic architecture of BD is controlled by many SNPs (9077 for HyB_BR; 9611 for BayesR) with small effects (the variance 0.0001*σ*_*g*_^2^), but just 3 SNPs with large effects (the variance 0.01*σ*_*g*_^2^). These numbers demonstrated the polygenic architecture of BD. On the contrary, for T1D, there was relatively smaller number of SNPs (3544 for HyB_BR; 2750 for BayesR) with small effects but many more SNPs (almost 200) with large effects. The proportion numbers from Fig. 8 also demonstrated this (in accordance with the results from Moser et al. [8]). Large proportion of SNPs with small effects (the variance 0.0001*σ*_*g*_^2^) controlled the polygenic architecture of the diseases BD (98.76 % for HyB_BR; 99.55 % for BayesR), CAD (97.31 % for HyB_BR; 96.8 % for BayesR), HT (96.96 % for HyB_BR; 98.09 % for BayesR), and T2D (95.14 % for HyB_BR; 97.79 % for BayesR). For these diseases, the mixture model of BayesR and HyB_BR did not have much advantage. However, relatively larger proportions of SNPs with moderate effects (the variance 0.001*σ*_*g*_^2^) existed for the traits RA (0.77 % for HyB_BR; 0.93 % for BayesR) and T1D(5.02 % for HyB_BR; 5.54 % for BayesR). For these two traits controlled by major genes, BayesR and HyB_BR gave substantially greater accuracy than GBLUP, which explained the results for prediction accuracy (Table 10).

**Table 11:** The number of SNPs in each proportion of four distributions estimated by BayesR, and HyB_BR on seven human diseases

Diseases	BayesR				HyB_BR			
	Pr[54]	Pr[2]	Pr[3]	Pr[4]	Pr[54]	Pr[2]	Pr[3]	Pr[4]
BD	282,843	9611	39	3	283,306	9077	110	3
CAD	289,491	6892	214	13	289,203	7211	183	13
CD	294,423	6878	269	9	294,463	6576	331	9
HT	286,152	8094	150	8	286,160	7993	243	8
RA	291,401	4172	275	42	290,420	5025	403	42
T1D	293,366	2607	54	200	292,523	3396	104	207
T2D	286,489	7972	173	7	288,365	5971	298	7

Compared with BayesR, HyB_BR detected the same number of SNPs with moderate variance (the variance 0.01 * *σ*_*g*_^2^) but appeared to systematically detect more SNPs in the proportion of small variance (the variance 0.0001 * *σ*_*g*_^2^), similar to the results observed for the comparison of BayesR and HyB_BR in in dairy cattle data (Table 8).

## Discussion

We have presented a novel and computationally efficient algorithm termed HyB_BR for simultaneous genomic prediction and QTL mapping. A pure EM algorithm was less accurate for some traits, while pure MCMC requires very long computation times. Therefore, HyB_BR implements the EM algorithm followed by a limited number of MCMC iterations. In this way, the algorithm takes advantage of the features of an EM algorithm (rapid convergence) and the higher accuracy from MCMC implementations in a hybrid scheme. Our accuracies of genomic prediction for complex traits in human and cattle from HyB_BR are almost identical to those from the full MCMC implementation of the Bayesian mixture model, with a 10 fold or greater reduction in computing time required.

For the pure MCMC algorithm, the burn-in stage can account for up to 50 % of the total running time. One of the key advantages of HyB_BR is that the EM module effectively replaces the burn-in cycles that are usually required for MCMC. Based on the starting point from EM (with very limited number of iterations; less than 500 iterations), the running time of HyB_BR can be much reduced.

The pure EM algorithm, EmBayesR [30] has been demonstrated to be much faster than BayesR, but had lower accuracy for some traits, particularly those with mutations of moderate to large effect. For example, when implemented on the trait fat% in dairy cattle, emBayesR had a decreased accuracy of 5 % ~ 7 % compared to BayesR. One possible explanation is that emBayesR shrinks SNP effects too much (shown in Table 8). This could be because the PEV that is used to account for the error of the effects of all the other SNPs while estimating the effect of the current SNP is only an approximation. The introduction of PEV correction is based on one observation: previous fast algorithm studies (especially Iterative conditional expectation algorithms) assumed the effects of the other SNPs were estimated perfectly while estimating the effect of the current SNP, leading to poor performance [30]. Therefore, EmBayesR and the EM part of HyB_BR allow for the errors in the effect of other SNPs and other location parameters by using the PEV. The calculation of the PEV from GBLUP is carried out before the iterations to estimate the effects of each SNP. And since the normal priors from GBLUP model do not allow for SNPs of moderate to large effects, such PEV calculation is an approximation and this may be one reason for loss of accuracy in the EM. To deal with this, HyB_BR further implements a small number of MCMC iterations to improve the outcome of pure EM steps.

HyB_BR has three advantages. First, as the size of genomic data increases, the computational efficiency of HyB_BR without burn-in stage (a small number of *O*(*mn*) iterations), is greater than BayesR by full MCMC. And when implemented with the speed-up scheme described in the methods, computational time can be reduced even further, by sampling a reduced set of SNPs in the MCMC module, apparently with no loss of accuracy (but critically the information from the SNPs that are not sampled remains in the posterior proportions of SNPs in each distribution). Second, the prediction accuracy of HyB_BR is comparable to BayesR in all cases including dairy cattle and human disease prediction shown in Tables 5, 6 and 7, and Table 10. Third, HyB_BR, like BayesR, is flexible with respect to the genetic architecture of complex traits. As shown in Tables 5, 6 and 7, HyB_BR performs well on four different complex traits, with architecture ranging from highly polygenic architecture to genetic architecture controlled by major genes. In addition to the prediction on the continuous quantitative traits of dairy cattle, the investigation on the risk prediction of seven case/control human diseases with binary 0/1 phenotypes shows HyB_BR and BayesR perform on this type of data, Table 10. Finally, the posterior probabilities of SNP having a nonzero effect from HyB_BR can be used for QTL mapping, Fig. 6.

Implementing genomic prediction methods with whole genome sequence data may improve the prediction accuracy and accelerate the discovery of causal variants. However, for this to occur, more computationally efficient genomic prediction algorithms are required. Compared with BayesR, the predicted time of HyB_BR on different number of markers with the same reference phenotypesis listed in Table 12. The time is estimated linearly on the number of markers and individuals. When the number of markers reaches 30 million (the number of variants discovered in the 1000 bull genomes project, Daetwyler et al. [28]), the running time of BayesR is around 34,170 h, which is impractical. On the contrary, on the same data with 30 million of variants, HyB_BR is predicted to require 2010 h. It may be possible to reduce this further by optimising the code even more. Therefore, as the size of genomic data increases, HyB_BR will remain feasible well beyond the point where the use of BayesR is impractical.

**Table 12:** The predicted computational time (in hours) of HyB_BR and BayesR on high density data with different number of variants and the same number of individuals (16,214)

	Different number of markers			
	800 K SNP panel	1 million	2 million	30 millions
BayesR	720 h	1139 h	2278 h	34,170 h
HyB_BR	42 h	67 h	134 h	2010 h

While HyB_BR performs well with computational efficiency and robust prediction accuracy, there are at least still two strategies that could be used to further improve efficiency. There is one key part of EM module that consumes running time and memory: the calculation of *tr*(**E**^− 1^**Z**_**i**_**Z**_**i**_^'^**E**^− 1^**PEV**) for each SNP in front of EM iterations. In detail, the calculation of *tr*(**E**^− 1^**Z**_**i**_**Z**_**i**_^'^**E**^− 1^**PEV**) requires the time complexity of $O\left(\frac{1}{2}m{n}^2\right)$, which accounts for almost 2/3 of the computational time required for the EM module even though the calculation is made in front of the EM iterations. Therefore, a future task is to implement a multi-threaded version to improve speed. The threshold of limiting the number of SNPs to be updated requires further study. Currently we define the threshold as **T**: if(*P*(*i*, 1) > 0.9), which is applicable for the current data. However, it’s uncertain whether or not such a threshold is suitable for other types of data.

HyB_BR has some features in common with other mixture methods such as BSLMM [6], and BOLT-LMM [25]. All of these methods declared the merit of computational efficiency with time complexity *O*(*mn*) but under different mixture models. In detail, BSLMM assumed a large proportion of SNPs with small effects (under BLUP models), while others had large effects (under Bayesian sparse regression models; the mixture of two normal priors). Due to limited number of SNPs implemented for MCMC sampling (large proportion of SNPs are under GBLUP models), BSLMM could be computationally efficient. However, compared with the mixture of four normal distributions by BayesR which provided great flexibility with respect genetic architecture, the flexibility of BSLMM with respect to different genetic architectures required further investigation. Another algorithm is BOLT-LMM, which has been developed mainly for the association studies. BOLT-LMM incorporated Bayesian mixture models to improve the power of GWAS with appealing outcomes. Instead of MCMC sampling, BOLT-LMM implemented iterative conditional expectation (ICE) algorithm on a mixture of two normal distributions to improve the computational speed with the approximated computational complexity *O*(*mn*). There could be three limitations with this method: 1) ICE algorithms did not account for the PEVs from all other SNP effects during the estimation of current SNP effect. On practical data sets, ICE could lead to the loss of prediction accuracy. BOLT-LMM introduced LD score regression technique to calibrate the prediction errors. However, since the calibrating factor was constant across all the SNPs (the prediction error variance regarding each SNP differs according to our equation (**E**^− 1^**Z**_**i**_**Z**_**i**_^'^**E**^− 1^**PEV**)), such calibration scheme seem not to be effective to solve the problem. 2) The leave-one-chromosome-out scheme implemented in BOLT-LMM might perform well for GWAS but not be suitable for simultaneous genomic prediction. 3) BOLT-LMM treated each SNP effect as a fixed effect for the association statistics. This combined with the stringent significance threshold for multiple testing, leaded to the over-estimation for SNP effects. Another efficient method for genomic prediction termed MultiBLUP [7] introduced SNPs clusters into BLUP models according to its adaptive algorithm. For each SNP class, the linear combination models (using genomic relationship matrix) similar to GBLUP were implemented. MultiBLUP has been demonstrated to be computationally efficient with robust prediction accuracy in the human data sets. However, when moved to dairy cattle genomic data sets, there is long Linkage disequilibrium (LD) between markers, which might be easily broken up by multiBLUP models.

## Conclusions

In summary, HyB_BR is a computationally efficient method for simultaneous genomic prediction, QTL mapping and inference of genetic architecture. The hybrid scheme of MCMC and EM decreases computational time by a factor of at least 10 fold with no reduction in prediction accuracy. The HyB_BR algorithm makes simultaneous genomic prediction, QTL mapping and inference of genetic architecture feasible in extremely large genomic data sets including whole genome sequence data.

## Additional files

Additional file 1: PEV calculation from GBLUP. (DOCX 16 kb) Additional file 2: Calculation of *P*(*i*, *k*). (DOCX 21 kb)

## References

[1] Meuwissen THE, Hayes BJ, Goddard ME (2001). Prediction of total genetic value using genome-wide dense marker maps. Genetics.

[2] Goddard ME, Hayes BJ (2009). Mapping genes for complex traits in domestic animals and their use in breeding programmes. Nat Rev Genet.

[3] Meuwissen T, Hayes B, Goddard M (2013). Accelerating improvement of livestock with genomic selection. Annu. Rev. Anim. Biosci.

[4] Yang J, Benyamin B, McEvoy BP, Gordon S, Henders AK, Nyholt DR, Madden PA, Heath AC, Martin NG, Montgomery GW (2010). Common SNPs explain a large proportion of the heritability for human height. Nat Genet.

[5] de los Campos G, Gianola D, Allison DB (2010). Predicting genetic predisposition in humans: the promise of whole-genome markers. Nat Rev Genet.

[6] Zhou X, Carbonetto P, Stephens M (2013). Polygenic modeling with Bayesian sparse linear mixed models. PLoS Genet.

[7] Speed D, Balding DJ (2014). MultiBLUP: improved SNP-based prediction for complex traits. Genome Res.

[8] Moser G, Lee SH, Hayes BJ, Goddard ME, Wray NR, Visscher PM (2015). Simultaneous discovery, estimation and prediction analysis of complex traits using a Bayesian mixture model. PLoS Genet.

[9] VanRaden PM (2008). Efficient methods to compute genomic predictions. J Dairy Sci.

[10] VanRaden PM, Null DJ, Sargolzaei M, Wiggans GR, Tooker ME, Cole JB, Sonstegard TS, Connor EE, Winters M, van Kaam JBCHM (2013). Genomic imputation and evaluation using high-density holstein genotypes. J Dairy Sci.

[11] Wolc A, Zhao HH, Arango J, Settar P, Fulton JE, O’Sullivan NP, Preisinger R, Stricker C, Habier D, Fernando RL (2015). Response and inbreeding from a genomic selection experiment in layer chickens. Genet Sel Evol.

[12] Gianola D (2013). Priors in whole-genome regression: the Bayesian alphabet returns. Genetics.

[13] Habier D, Fernando RL, Kizilkaya K, Garrick DJ (2011). Extension of the bayesian alphabet for genomic selection. BMC Bioinf.

[14] Erbe M, Hayes BJ, Matukumalli LK, Goswami S, Bowman PJ, Reich CM, Mason BA, Goddard ME (2012). Improving accuracy of genomic predictions within and between dairy cattle breeds with imputed high-density single nucleotide polymorphism panels. J Dairy Sci.

[15] Kemper KE, Reich CM, Bowman PJ, vander Jagt CJ, Chamberlain AJ, Mason BA, Hayes BJ, Goddard ME (2015). Improved precision of QTL mapping using a nonlinear Bayesian method in a multi-breed population leads to greater accuracy of across-breed genomic predictions. Genet Sel Evol.

[16] Grisart B, Coppieters W, Farnir F, Karim LCF, Berzi P, Cambisano N, Mni M, Reid S, Simon P (2002). Positional candidate cloning of a QTL in dairy cattle: identification of a missense mutation in the bovine DGAT1 gene with major effect on milk yield and composition. Genome Res.

[17] Ng-Kwai-Hang K (1997). A review of the relationship between milk protein polymorphism and milk composition/milk production. Proceedings of the international dairy federation seminar: 25–27 febuary, 1997 1997; palmerston north, New Zealand.

[18] Blott S, Kim J-J, Moisio S, Schmidt-Küntzel A, Cornet A, Berzi P, Cambisano N, Ford C, Grisart B, Johnson D (2003). Molecular dissection of a quantitative trait locus: a phenylalanine-to-tyrosine substitution in the transmembrane domain of the bovine growth hormone receptor is associated with a major effect on milk yield and composition. Genetics.

[19] Wang X, Wurmser C, Pausch H, Jung S, Reinhardt F, Tetens J, Thaller G, Fries R (2012). Identification and dissection of four major QTL affecting milk Fat content in the German holstein-friesian population. PLoS One.

[20] Zhang Z, Ersoz E, Lai C-Q, Todhunter RJ, Tiwari HK, Gore MA, Bradbury PJ, Yu J, Arnett DK, Ordovas JM (2010). Mixed linear model approach adapted for genome-wide association studies. Nat Genet.

[21] Lippert C, Listgarten J, Liu Y, Kadie CM, Davidson RI, Heckerman D (2011). FaST linear mixed models for genome-wide association studies. Nat Meth.

[22] Zhou X, Stephens M (2012). Genome-wide efficient mixed-model analysis for association studies. Nat Genet.

[23] Listgarten J, Lippert C, Kadie CM, Davidson RI, Eskin E, Heckerman D (2012). Improved linear mixed models for genome-wide association studies. Nat Meth.

[24] Yang J, Zaitlen NA, Goddard ME, Visscher PM, Price AL (2014). Advantages and pitfalls in the application of mixed-model association methods. Nat Genet.

[25] Loh P-R, Tucker G, Bulik-Sullivan BK, Vilhjalmsson BJ, Finucane HK, Salem RM, Chasman DI, Ridker PM, Neale BM, Berger B (2015). Efficient Bayesian mixed-model analysis increases association power in large cohorts. Nat Genet.

[26] Wood AR, Esko T, Yang J, Vedantam S, Pers TH, Gustafsson S, Chu AY, Estrada K, Luan J, Kutalik Z (2014). Defining the role of common variation in the genomic and biological architecture of adult human height. Nat Genet.

[27] The Wellcome Trust Case Control Consortium (2007). Genome-wide association study of 14,000 cases of seven common diseases and 3,000 shared controls. Nature.

[28] Daetwyler HD, Capitan A, Pausch H, Stothard P, van Binsbergen R, Brondum RF, Liao X, Djari A, Rodriguez SC, Grohs C (2014). Whole-genome sequencing of 234 bulls facilitates mapping of monogenic and complex traits in cattle. Nat Genet.

[29] Calus MPL (2014). Right-hand-side updating for fast computing of genomic breeding values. Genet Sel Evol.

[30] Wang T, Chen Y-PP, Goddard ME, Meuwissen THE, Kemper KE, Hayes BJ (2015). A computationally efficient algorithm for genomic prediction using a Bayesian model. Genet Sel Evol.

[31] Meuwissen THE, Solberg TR, Shepherd R, Woolliams JA (2009). A fast algorithm for BayesB type of prediction of genome-wide estimates of genetic value. Genet Sel Evol.

[32] Yu X, Meuwissen THE (2011). Using the pareto principle in genome-wide breeding value estimation. Genet Sel Evol.

[33] Shepherd RK, Meuwissen THE, Woolliams JA (2010). Genomic selection and complex trait prediction using a fast EM algorithm applied to genome-wide markers. BMC Bioinf.

[34] Hayashi T, Iwata H (2010). EM algorithm for Bayesian estimation of genomic breeding values. BMC Genet.

[35] Sun X, Qu L, Garrick DJ, Dekkers JCM, Fernando RL (2012). A fast EM algorithm for BayesA-like prediction of genomic breeding values. PLoS One.

[36] Garrick D, Taylor J, Fernando R (2009). Deregressing estimated breeding values and weighting information for genomic regression analyses. Genet Sel Evol.

[37] Henderson C (1984). Application of linear models in animal breeding.

[38] Browning BL, Browning SR (2009). A unified approach to genotype imputation and haplotype-phase inference for large data sets of trios and unrelated individuals. Am J Hum Genet.

[39] Haile-Mariam M, Bowman PJ, Pryce JE (2013). Genetic analyses of fertility and predictor traits in Holstein herds with low and high mean calving intervals and in Jersey herds. J Dairy Sci.

[40] Haile-Mariam M, Pryce JE, Schrooten C, Hayes BJ (2015). Including overseas performance information in genomic evaluations of Australian dairy cattle. J Dairy Sci.

[41] Gilmour A, Cullis B, Welham S, Thompson R (2002). ASReml reference manual 2nd edition.

[42] Lee Sang H, Wray Naomi R, Goddard Michael E, Visscher Peter M (2011). Estimating missing heritability for disease from genome-wide association studies. Am J Human Gen.

[43] Purcell S, Neale B, Todd-Brown K, Thomas L, Ferreira MAR, Bender D, Maller J, Sklar P, de Bakker PIW, Daly MJ (2007). PLINK: a tool Set for whole-genome association and population-based linkage analyses. Am J Hum Genet.

[44] Yang J, Lee SH, Goddard ME, Visscher PM (2011). GCTA: a tool for genome-wide complex trait analysis. Am J Hum Genet.

[45] Wray NR, Yang J, Goddard ME, Visscher PM (2010). The genetic interpretation of area under the ROC curve in genomic profiling. PLoS Genet.

[46] Hozé C, Fritz S, Phocas F, Boichard D, Ducrocq V, Croiseau P (2014). Efficiency of multi-breed genomic selection for dairy cattle breeds with different sizes of reference population. J Dairy Sci.

[47] Chamberlain AJ, Vander Jagt CJ, Hayes BJ, Khansefid M, Marett LC, Millen CA, Nguyen TTT, Goddard ME (2015). Extensive variation between tissues in allele specific expression in an outbred mammal. BMC Genomics.

[48] Sanders K, Bennewitz J, Reinsch N, Thaller G, Prinzenberg EM, Kühn C, Kalm E (2006). Characterization of the DGAT1 mutations and the CSN1S1 promoter in the German angeln dairy cattle population. J Dairy Sci.

[49] Raven L-A, Cocks BG, Kemper KE, Chamberlain AJ, Jagt CJ, Goddard ME, Hayes BJ (2015). Targeted imputation of sequence variants and gene expression profiling identifies twelve candidate genes associated with lactation volume, composition and calving interval in dairy cattle. Mamm Genome.

[50] Pryce JE, Bolormaa S, Chamberlain AJ, Bowman PJ, Savin K, Goddard ME, Hayes BJ (2010). A validated genome-wide association study in 2 dairy cattle breeds for milk production and fertility traits using variable length haplotypes. J Dairy Sci.

[51] Cole JB, Wiggans GR, Ma L, Sonstegard TS, Lawlor TJ, Crooker BA, Van Tassell CP, Yang J, Wang S, Matukumalli LK (2011). Genome-wide association analysis of thirty one production, health, reproduction and body conformation traits in contemporary U.S. Holstein cows. BMC Genomics.

[52] McClure MC, Morsci NS, Schnabel RD, Kim JW, Yao P, Rolf MM, McKay SD, Gregg SJ, Chapple RH, Northcutt SL (2010). A genome scan for quantitative trait loci influencing carcass, post-natal growth and reproductive traits in commercial Angus cattle. Anim Genet.

[53] Wickramasinghe S, Hua S, Rincon G, Islas-Trejo A, German JB, Lebrilla CB, Medrano JF (2011). Transcriptome profiling of bovine milk oligosaccharide metabolism genes using RNA-sequencing. PLoS One.

[54] Consortium TGP (2010). A map of human genome variation from population-scale sequencing. Nature.

