# Supplementary material for: A hybrid expectation maximisation and MCMC sampling algorithm to implement Bayesian mixture model based genomic prediction and QTL mapping
Source: BMC Genomics. 2016 Sep 21;17:744. doi: 10.1186/s12864-016-3082-7 (PMC5031345; doi:10.1186/s12864-016-3082-7)
Supplement: Additional file 1: — PEV calculation from GBLUP. (DOCX 16 kb) [file 12864_2016_3082_MOESM1_ESM.docx]

**Additional file 1 - PEV calculation from GBLUP**

The prediction error variance $PEV(\mathbf{e}^{\boldsymbol{*}})$ is derived using GBLUP under the data model as follows:

$\mathbf{y}=\boldsymbol{X\beta}+\mathbf{u}^{\boldsymbol{*}}+\mathbf{Wv}+\mathbf{e}$,

here, $\mathbf{u}^{\boldsymbol{*}}\mathbf{=Zg,} \mathbf{u}^{\boldsymbol{*}}\sim N(0,\mathbf{G}\sigma_{g}^{2})$. In theory, $\mathbf{e}^{\boldsymbol{*}}=\mathbf{e+}\mathbf{Z}_{i}g_{i}$, therefore, $PEV(\mathbf{e}^{\boldsymbol{*}})\neq PEV(\mathbf{e})$. However, the difference should be small since the estimated effect from GBLUP model is shrunk to very small number. Therefore,$PEV(\mathbf{e})$ could be treated as the approximated calculation of $PEV(\mathbf{e}^{\boldsymbol{*}})$.

The calculation of $PEV(\mathbf{e})$ is described as follows:

$$PEV(e)=Var\left( \mathbf{e}-\hat{\mathbf{e}} \right)=\mathrm{Var}(\mathbf{e-}\mathbf{R}^{\mathbf{'}}\mathbf{V}^{\mathbf{-1}}\mathbf{y}\boldsymbol{)}$$

where, $\mathbf{V}$ is the variance of phenotype $\mathbf{y}$; $\mathbf{V=}\mathbf{G}^{\mathbf{'}}\mathbf{+Q+R}$ ($\mathbf{G}^{\mathbf{'}}$ is the variance and co-variance matrix of $\mathbf{u}$ by SNPs,$\mathbf{Q}$ is the variance and co-variance matrix by polygenes, **R** is the variance and co-variance error matrix).

Therefore,

$\mathrm{PEV}\left( \mathbf{e} \right)=Var\left( \mathbf{e-R}\mathbf{V}^{\mathbf{-1}}\mathbf{y} \right)$

$\boldsymbol{=}\mathrm{Var}\left( \mathbf{e} \right)+Var\left( \mathbf{R}\mathbf{V}^{\mathbf{-1}}\mathbf{y} \right)-2cov\left( \mathbf{u,R}\mathbf{V}^{\mathbf{-1}}\mathbf{y} \right)$

$=\mathbf{R}+\mathbf{R}\mathbf{V}^{\mathbf{-1}}\mathrm{Var}\left( \mathbf{y} \right)\mathbf{V}^{\mathbf{-1}}\mathbf{R}\boldsymbol{-}2\mathbf{R}\mathbf{V}^{\mathbf{-1}}\mathbf{cov}\left( \mathbf{u,y} \right)$

$\mathbf{=R}\boldsymbol{+}\mathbf{R}\mathbf{V}^{\mathbf{-1}}\mathbf{R}\boldsymbol{-2}\mathbf{R}\mathbf{V}^{\mathbf{-1}}\mathbf{R}$

$\mathbf{=R}\boldsymbol{-}\mathbf{R}\mathbf{V}^{\mathbf{-1}}\mathbf{R}$

$\boldsymbol{=}\left[ \left( \mathbf{Q+}\mathbf{G}^{\mathbf{'}} \right)^{\boldsymbol{-1}}\boldsymbol{+}\mathbf{R}^{\mathbf{-1}} \right]^{\boldsymbol{-1}}$

Substitute it with $\mathbf{G}^{\mathbf{'}}\mathbf{=G}\sigma_{g}^{2}\mathbf{, R=E}\sigma_{e}^{2}, \mathbf{Q=A}\sigma_{a}^{2}$ , we get:

${\mathrm{PEV}= (\mathbf{E}^{-1}\sigma_{e}^{-2}+{(\mathbf{G}\sigma_{g}^{2}+\mathbf{A}\sigma_{a}^{2})}^{-1})}^{-1}$ (S1)

Afterwards, such PEV matrix will be used in Appendix 2.
