# Supplementary material for: A hybrid expectation maximisation and MCMC sampling algorithm to implement Bayesian mixture model based genomic prediction and QTL mapping
Source: BMC Genomics. 2016 Sep 21;17:744. doi: 10.1186/s12864-016-3082-7 (PMC5031345; doi:10.1186/s12864-016-3082-7)
Supplement: Additional file 2: — Calculation of P(i, k). (DOCX 21 kb) [file 12864_2016_3082_MOESM2_ESM.docx]

**Additional file 2 - Calculation of** $\boldsymbol{P}\left( \boldsymbol{i,k} \right)$

The parameter $P\left( i,k \right)$ defines the probability that each SNP $i$ follows in the $k^{th}$ normal distribution ($k=1,2,3,4)$ conditional on the data. An important part of the EM algorithm for the mixture BayesR model is estimating:

$P\left( i,k \right)=p(b\left( i,k \right)=1|\mathbf{y},{Pr}_{k},\sigma_{e}^{2},\boldsymbol{\beta,v})$.

Suppressing the parameters not involving $b\left( i,k \right)$, we get:

$P\left( i,k \right)=p\left( b\left( i,k \right)=1 | \mathbf{y},{Pr}_{k},\sigma_{e}^{2},\boldsymbol{\beta,v} \right)$

$\propto p(b\left( i,k \right)=1|\mathbf{y},{Pr}_{k})$

$\propto p\left( \mathbf{y} | b\left( i,k \right)=1 \right)p(b\left( i,k \right)=1|{Pr}_{k})$

Under the model (1a), we introduce the “missing data” $e^{*}=\mathbf{y}-X\boldsymbol{\beta}-\mathbf{u}-\mathbf{W}\mathbf{v=}\mathbf{Z}_{\mathbf{i}}g_{i}+e$. Therefore, $e^{*}\sim N(0,$ $\mathbf{H}_{\mathbf{k}}$), with $\mathbf{H}_{\mathbf{k}}\boldsymbol{=}\mathbf{Z}_{\mathbf{i}}\mathbf{Z}_{\mathbf{i}}'\sigma^{2}[k]+\mathbf{E}\sigma_{e}^{2}$. The posterior expression of $P_{ik}$ can be rewritten as:

$$P\left( i,k \right)\propto p\left( e^{*} | b\left( i,k \right)=1 \right)p(b\left( i,k \right)=1|{Pr}_{k})$$

$$logP\left( i,k \right)=logp\left( e^{*} | b\left( i,k \right)=1 \right)+logp(b\left( i,k \right)=1|{Pr}_{k})$$

$=-\frac{1}{2}(\log\left| \mathbf{H}_{k} \right|+(e^{*})'{\mathbf{H}_{\mathbf{k}}}^{-1} e^{*}+log{Pr}_{k}$)

Take expectation of $logP_{ik}$ regarding the missing data $e^{*}$ as follows:

$E_{e^{*}}logP\left( i,k \right)=-\frac{1}{2}(\log\left| \mathbf{H}_{k} \right|+(e^{*})'{\mathbf{H}_{\mathbf{k}}}^{-1} e^{*}+tr({\mathbf{H}_{\mathbf{k}}}^{-1}PEV(e^{*}))+log{Pr}_{k}$). (S2)

Here,$\mathbf{H}_{\mathbf{k}}\boldsymbol{=}\mathbf{Z}_{\mathbf{i}}\mathbf{Z}_{\mathbf{i}}'\sigma^{2}[k]+\mathbf{E}\sigma_{e}^{2}$, $PEV(e^{*})$ is estimated in the Appendix 1. According to the Woodbury Identity theory, the calculation of the equation ${\mathbf{H}_{\mathbf{k}}}^{-1}$ and $log\left| \mathbf{H}_{\mathbf{k}} \right|$ can be simplified as

$${\mathbf{H}_{\mathbf{k}}}^{\mathbf{-1}}\mathbf{=}\left( \mathbf{Z}_{\mathbf{i}}\mathbf{Z}_{\mathbf{i}}^{\mathbf{'}}\sigma_{i}^{2}[k]\mathbf{+E}\boldsymbol{\sigma}_{\mathbf{e}}^{\mathbf{2}} \right)^{\mathbf{-1}}\mathbf{=}\boldsymbol{\sigma}_{\mathbf{e}}^{\mathbf{-2}}\left( \mathbf{E}^{\mathbf{-1}}\mathbf{-}\frac{\mathbf{E}^{\mathbf{-1}}\mathbf{Z}_{\mathbf{i}}\mathbf{Z}_{\mathbf{i}}^{\mathbf{'}}\mathbf{E}^{\mathbf{-1}}\sigma_{i}^{2}[k]}{\sigma_{i}^{2}[k]{\mathbf{Z}_{\mathbf{i}}^{\mathbf{'}}\mathbf{E}^{\mathbf{-1}}\mathbf{Z}}_{\mathbf{i}}\mathbf{+}\boldsymbol{\sigma}_{\mathbf{e}}^{\mathbf{2}}} \right)$$

$$log\left| \mathbf{H}_{\mathbf{k}} \right|\boldsymbol{=}\left( n-1 \right)\mathrm{lo}g \sigma_{e}^{2}+log|\mathbf{E}|+log\left( \sigma_{i}^{2}[k]\mathbf{Z}_{\mathbf{i}}^{\boldsymbol{'}}{\mathbf{E}^{-1}\mathbf{Z}}_{\mathbf{i}}\boldsymbol{+}\sigma_{e}^{2} \right)$$

Therefore, the equation (S2) can be simplified as: $E_{e^{*}}logP\left( i,k \right)=log\Pr_{k}-\frac{1}{2}\left\{ \left( n-1 \right)\mathrm{lo}g \sigma_{e}^{2}+\log\left| \mathbf{E} \right|+\log\left( \sigma_{i}^{2}[k]\mathbf{Z}_{\mathbf{i}}^{\boldsymbol{'}}\mathbf{Z}_{\mathbf{i}}\boldsymbol{+}\sigma_{e}^{2} \right) \right\}$ $-\frac{1}{2}\left\{ \left( \left( e^{*} \right)^{'\mathbf{E}^{\mathbf{-1}}e^{*}} \right)\sigma_{e}^{-2}\mathbf{-}{\left( \left( e^{*} \right)^{'\mathbf{E}^{\mathbf{-1}}\mathbf{Z}_{\mathbf{i}}} \right)^{2}\sigma_{i}^{2}\left[ k \right]\sigma_{e}^{-2}}/\left( \sigma_{i}^{2}\left[ k \right]\mathbf{Z}_{\mathbf{i}}^{\boldsymbol{'}}\mathbf{E}^{\mathbf{-1}}\mathbf{Z}_{\mathbf{i}}\boldsymbol{+}\sigma_{e}^{2} \right) \right\}$ $-\frac{1}{2}\left\{ \mathrm{tr}\left( \mathbf{E}^{\mathbf{-1}}PEV(e^{*}) \right)\sigma_{e}^{-2}-{\mathrm{tr}\left( \mathbf{E}^{\mathbf{-1}}\mathbf{Z}_{\mathbf{i}}\mathbf{Z}_{\mathbf{i}}^{\boldsymbol{'}}\mathbf{E}^{\mathbf{-1}}PEV(e^{*}) \right)\sigma_{i}^{2}[k]\sigma_{e}^{-2}}/\left( \sigma_{i}^{2}[k]\mathbf{Z}_{\mathbf{i}}^{\boldsymbol{'}}\mathbf{E}^{\mathbf{-1}}\mathbf{Z}_{\mathbf{i}}\boldsymbol{+}\sigma_{e}^{2} \right) \right\}$ (3)

Then, $P\left( i,k \right)=\frac{exp(E_{e^{*}}logP\left( i,k \right))}{\sum_{k=1}^{4} exp(E_{e^{*}}logP\left( i,k \right))}$*.* (4)
